# Supplementary material for: Age, health and other factors associated with return to work for those engaging with a welfare-to-work initiative: a cohort study of administrative data from the UK’s Work Programme
Source: BMJ Open. 2018 Oct 27;8(10):e024938. doi: 10.1136/bmjopen-2018-024938 (PMC6224745; doi:10.1136/bmjopen-2018-024938)
Supplement: Author's manuscript [file bmjopen-2018-024938.draft_revisions.pdf]

BMJ Open is committed to open peer review. As part of this commitment we make the peer review history of every article we publish publicly available.

When an article is published we post the peer reviewers' comments and the authors' responses online. We also post the versions of the paper that were used during peer review. These are the versions that the peer review comments apply to.

The versions of the paper that follow are the versions that were submitted during the peer review process. They are not the versions of record or the final published versions. They should not be cited or distributed as the published version of this manuscript.

BMJ Open is an open access journal and the full, final, typeset and author-corrected version of record of the manuscript is available on our site with no access controls, subscription charges or pay-per-view fees (http://bmjopen.bmj.com).

If you have any questions on BMJ Open's open peer review process please email
info.bmjopen@bmj.com

BMJ Open

Age, health and other factors associated with return to work for those engaging with a welfare-to-work initiative: A cohort study of administrative data from the UK's Work Programme

Journal:	*BMJ Open*
Manuscript ID	bmjopen-2018-024938
Article Type:	Research
Date Submitted by the Author:	21-Jun-2018

Complete List of Authors:	Brown, Judith; University of Glasgow, Institute of Health and Wellbeing<br>Katikireddi, Srinivasa; University of Glasgow School of Life Sciences, MRC/CSO Social & Public Health Sciences Unit<br>Leyland, Alastair; MRC/CSO Social and Public Health Sciences Unit, University of Glasgow,<br>McQuaid, Ronald; University of Stirling, Management Work and Organisation<br>Frank, John; University of Edinburgh, The Usher Institute of Population Health Sciences and Informatics, College of Medicine and Veterinary Medicine,<br>Macdonald, Ewan; University of Glasgow, Institute of Health and Wellbeing

Keywords:	PUBLIC HEALTH, Ageing, Health, Unemployment, Return to work, Vocational rehabilitation

SCHOLARONE™
Manuscripts

For peer review only

Age, health and other factors associated with return to work for those engaging with a welfare-to-work initiative: A cohort study of administrative data from the UK's Work Programme

Judith Brown,[1] Srinivasa Vittal Katikireddi,[2] Alastair H Leyland,[2] Ronald W McQuaid,[3] John Frank,[4] Ewan B Macdonald[1]

[1] Healthy Working Lives Group, Institute of Health and Wellbeing, College of Medical, Veterinary and Life Sciences, University of Glasgow, Glasgow, G12 8RZ

[2] MRC /CSO Social and Public Health Sciences Unit, Institute of Health and Wellbeing, College of Medical, Veterinary and Life Sciences, University of Glasgow, Glasgow, G12 8RZ

[3] Management Work and Organisation, University of Stirling, Stirling, FK9 4LA

[4]Scottish Collaboration for Public Health Research and Policy (SCPHRP), University of Edinburgh, 20 West Richmond Street, Edinburgh, EH8 9DX

Correspondence to: Dr Judith Brown

Judith.Brown@glasgow.ac.uk

Key words: ageing; health; unemployment; return to work; vocational rehabilitation

Word Count: 4825

For peer review only

ABSTRACT

Objectives

To investigate the role of individual factors (including age, health and personal circumstances) and external factors associated with clients having a job start while engaging with the Work Programme and variations by benefit type.

Setting

The UK Government's main return to work initiative (The Work Programme) in Scotland.

Design

Piecewise Poisson regression to calculate incident rate ratios (IRR) using administrative data from 2013 to 2016 to identify factors associated with job start.

Participants

4322 Employment Support Allowance (ESA) clients not in work due to poor health and 8996 Jobseeker's Allowance (JSA) clients, aged 18–64 years, referred to the Work Programme between April 2013 and July 2014.

Main outcome measures

Starting a job and the time to first job start after entering the Work Programme.

Results

JSA clients (62%) were more likely to return to work than ESA clients (20%). There is a strong negative relationship between age and the predicted probability of having a job start during the two-year engagement with the programme for both JSA and ESA clients. JSA clients were most likely to have their first job start in their first three months on the programme while for ESA clients the predicted probability of having a first job start was fairly constant over the 2 years. Health, including the number of

For peer review only - http://bmjopen.bmj.com/site/about/guidelines.xhtml

health conditions disclosed by clients, and other individual and external factors associated with job starts were determined for both groups.

Conclusions

Age plays an important role in influencing return to work, however important potentially modifiable factors include the length of unemployment, training and education, the management of multimorbidity, and the individual's perception of the likelihood of job start. Future welfare-to-work programmes could be improved to enable more older people realise the potential health benefits of return to work.

For peer review only

For peer review only - http://bmjopen.bmj.com/site/about/guidelines.xhtml

Article Summary

Strength and limitations of study

- This is the first study to explore the role of individual age, health and other factors in returning to work, using large-scale administrative data collected over two years in the Work Programme.
- The use of administrative data (rather than survey data as in previous evaluations) limited loss to follow up, however the analysis relies on routine operational data with limited health diagnostic and severity information available.
- JSA and ESA clients were analysed separately showing important differences in return to work between the two client groups (by definition with and without an illness, health condition or disability that makes it difficult to work, although many JSA clients disclosed health conditions). As new programmes will potentially cater for JSA and ESA clients separately, this further illustrates the usefulness of our study analysing the two client groups separately.
- Other studies which have investigated the role of age have used one single age category (50+) or 5-year age categories. This study had modelled age as a continuous variable (1406 JSA clients and 1322 ESA clients aged 50 and over) to better understand the relationship between age and RTW.

For peer review only

For peer review only - http://bmjopen.bmj.com/site/about/guidelines.xhtml

INTRODUCTION

Labour market participation is an important determinant of health and health inequalities, with efforts to increase paid employment thought by policymakers to improve health.[1-5] However, health status also inhibits returning to work from being unemployed, especially for those with multiple health problems.[6-9] In addition, age is closely connected to both health status and other difficulties in returning to work, with employment rates for the working age population declining sharply from over 80% of 50 year olds in the UK, to around 60% of 60 year-olds with a steeper decline at older ages.[10] This paper uses a unique database to analyse the likelihood of unemployed people on the government's Work Programme (WP) returning to work. Specifically it considers the role of age when comparing those with an illness, health condition or disability that makes it difficult to work (those receiving the Employment and Support Allowance) and others on the programme (those receiving Jobseeker's Allowance).

Due to the ageing population, older workers increasingly make up a greater proportion of the workforce in most economically developed countries.[11] Thus recruiting and retaining older workers, and encouraging and enabling more people to work for longer, is a policy priority for governments and employers.[11 12] For instance, from 2014 to 2024 the UK will have 200,000 fewer people aged 16 to 49 and 3.2 million more people aged 50 to state pension age, but the latter with a lower average employment rate.[13]

Older workers face significant barriers in the labour market and are less likely to regain employment after job loss and are at increased risk of chronic health conditions

For peer review only - http://bmjopen.bmj.com/site/about/guidelines.xhtml

which contribute to job loss and may make re-employment difficult.[14-17] In addition, this age group may encounter barriers and factors that interact with their health including direct and indirect age discrimination,[18-20] skills gaps (especially in IT),[19] caring responsibilities, e.g. for grandchildren or elderly parents,[21] and the lack of flexible working opportunities.[22] Some individuals may experience multiple interacting or overlapping disadvantages which may be difficult to resolve in isolation.[8 23] For older workers (in this study refers to those aged 50 to 64) there are added difficulties of separating the impact of biological ageing from the impacts of unemployment and health selection effects (for example, ill health leading to early voluntary retirement).[5]

The WP was the UK Government's flagship welfare-to-work initiative to help those more detached from the labour market to enter employment and reduce the time people spent on benefits. It was launched throughout Great Britain in June 2011 as part of a sweeping programme of welfare reforms with final referrals in March 2017.[24] It required more people to either seek work or to undertake some form of work-related activity as a condition of receiving benefit.[25] The two-year programme was delivered by a range of private, public and voluntary sector organisations across eighteen regions. The Department for Work and Pensions (DWP) paid two or more prime contractors to provide directly or indirectly (through sub-contractors) support to unemployed job seekers in each region (delivered as a 'black box' approach where much of the control over services provided was wielded by the contractor). One innovatory feature was that payments were by results with most payments being after a participant had sustained employment (although not necessary

For peer review only - http://bmjopen.bmj.com/site/about/guidelines.xhtml

with the same employer) for a minimum time, rather than payments being mainly for job seekers participating or entering employment.

The unemployed including those out of work due to health reasons were required to participate in the WP, and others were able to volunteer to use the services depending on their circumstances. The WP supported two main groups of benefit claimants: Jobseeker's Allowance (JSA) clients and Employment and Support Allowance (ESA) clients. JSA is a benefit for people who are unemployed but capable of work, usually paid to unemployed people if all of the following apply: they are aged 18 to State Pension age, not in full-time education, living in Great Britain, available for work, actively seeking work and work on average less than 16 hours per week.[26] ESA is a benefit for people who have an illness, health condition or disability that makes it difficult to work and they will have undergone a Work Capability Assessment.[27] Claimants may get ESA if their illness or disability affects their ability to work and if they are: under State Pension age, not getting Statutory Sick Pay or Statutory Maternity Pay and have not gone back to work, and are not getting Jobseeker's Allowance.

One of the most extensive reports from the national WP evaluation to investigate factors influencing return to work (RTW) is a telephone survey of 4700 clients and a follow up survey of 1800 of the same clients.[8] It found that after two years on the programme, 67% of people were still not in work, and were more likely to be male, older than 55, have health conditions, few qualifications, and no recent work experience. Because having a health condition or disability was shown to be an important factor we believe there is a need to investigate ESA clients separately since

For peer review only

people with long-term health conditions and disabilities experience disproportionately lower employment rates. Data from 2015 show a significant gap in the employment rates of disabled (48%) and non-disabled people (80%),[28] and while the UK Government is committed to seeing one million more people in work over the next ten years,[29] if there is to be any chance of achieving this, then there must be a focus on improving outcomes for the over 50s and for those with disabilities in these new schemes. Furthermore, the evaluation investigated clients by age categories (18-24, 25-34, 35-44, 45-54, 55-59 & 60+) and for some analyses grouped clients aged 50+ into one group. The National Audit report of the WP did investigate JSA clients (termed easier-to-help) and ESA clients (termed harder-to-help) separately but they only reported on performance in getting people into work and not on the factors associated with RTW.[25] AgeUK reports divided over 50s into three age categories (50-54, 55-59, 60+) and suggested that low job performance is not caused by a higher incidence of disability or health conditions but rather age itself is the main barrier to work.[30 31]

In order to better understand all clients aged 50 and above we investigated individual factors, personal circumstances and external factors associated with RTW of JSA and ESA clients separately, treating age as a continuous variable. The aim of this study was to answer the following research questions:

1. What is the relationship between age and returning to work for JSA and ESA clients engaged with the WP?
2. How does the likelihood of returning to work change over the period of their participation in the WP for JSA and ESA clients?

For peer review only - http://bmjopen.bmj.com/site/about/guidelines.xhtml

3. What other factors, including health, are associated with RTW for JSA and ESA clients?

For peer review only

For peer review only - http://bmjopen.bmj.com/site/about/guidelines.xhtml

METHODS

Description of cohort

'Supporting Older people into Employment' (SOPIE) is a mixed methods longitudinal study involving a collaboration between academics, a major WP provider (Ingeus) and the UK DWP. Full details on the study, including sample size, can be found in the protocol paper.[32] The study population was all clients who entered the Ingeus WP in Scotland between the 1st April 2013 and the 31st July 2014 (14265 clients). After data cleaning the SOPIE cohort totalled 13318 clients. The 947 clients were removed from the study population as they: had significant missing baseline data including age (n=693); it was not able to generate datazone from postcode (n=100); were aged 16 to 17 (n=7); had lengths of unemployment which were not possible, for example a 20 year old with 10 years of unemployment history (n=147). The cohort was followed up longitudinally for the two years they engaged with Ingeus on the WP.

Variables

After referral to the WP clients completed a baseline face-to-face assessment with an employment advisor. The individual factors collected in the baseline assessment and used in this study were: age, gender, length of unemployment prior to the WP, highest qualification, ethnicity, whether the client had health concerns which they believed would affect their ability to work, number of health conditions disclosed to advisor, client perception of likelihood of job start, and personal circumstances (caring responsibility other than children, housing status, parental status). Ethnicity was recoded as white British and all other due to sample size. The number of health conditions disclosed by clients were categorised into 0, 1, 2 and 3 or more for JSA clients and 0/1, 2, 3, 4 and 5 or more for ESA clients. As expected ESA clients

For peer review only - http://bmjopen.bmj.com/site/about/guidelines.xhtml

disclosed a greater number of health conditions. 121 (2.8%) ESA clients disclosed no health conditions. This may have been a coding error or the client may have decided not to disclose their health condition to their advisor hence we coded 0 and 1 disclosed health conditions together (see Table 1).

Analyses of external factors were conducted using datazones. The 6976 datazones in Scotland have populations of between 500 and 1,000 household residents,[33] and Ingeus determined the datazone from the client's postcode. For each client the research team added the 2016 Scottish Index of Multiple Deprivation (SIMD) decile and the Scottish Government 8 fold urban rural classification (large urban areas, other urban areas, accessible small towns, remote small towns, very remote small towns, accessible rural, remote rural, very remote rural). The SIMD ranks datazones from the most (number 1) to the least deprived.[34 35]

Outcome measure

The primary outcome measures were: the client starting a job; and the length of time from beginning the WP to their first job start (months).

Statistical analysis

To address the three research questions a mixture of descriptive statistics and regression analyses were used. All analyses were stratified by benefit type (JSA and ESA clients) given the large differences in RTW between the two groups. Counts and percentages were used to summarise categorical variables. The associations between benefit type and all the study variables were analysed using chi-squared tests. Cox's proportional hazards models were initially used to determine the hazard ratios of

For peer review only - http://bmjopen.bmj.com/site/about/guidelines.xhtml

clients returning to work but the proportional hazards assumption was not met. We therefore used Poisson regression with fractional polynomials to model age as a continuous variable with a flexible functional form to predict the probability of having a job start.[36] Split times used in the models were as follows: 0 to 3 months, 3 to 6 months, 6 to 9 months, 9 to 12 months and 12 to 24 months (due to sample size a three month average probability is shown for the 12 to 24 month time period).

Unadjusted and adjusted incident rate ratios (IRR) and 95% confidence intervals (95% CI) were calculated to examine the associations between job start (RTW) and individual, personal and external factors. The unadjusted models contained age and gender and the adjusted model contained all the variables in the study. Overall differences between the categories are reported and for ordered variables (length of unemployment, highest qualification, number of health conditions disclosed to advisor, SIMD) a linear trend across categories was also determined. Predicted probabilities of RTW were estimated using post-estimation commands following regression modelling, with illustrative results shown for people aged 25 and 50 years when appropriate. All analyses were carried out using Stata version 14.

Patient/client involvement

No patients/clients were involved in developing the hypothesis, the specific aims or the research questions, nor were they involved in developing plans for design or implementation of the study. No patients were involved in the interpretation of study results or write up of the manuscript. Clients did provide feedback on emerging findings at yearly stakeholder meetings and a final study symposium.

For peer review only

RESULTS

Cohort demographics and job start

Of the SOPIE cohort of 13318 clients, 8996 (68%) were claiming JSA and 4322 (32%) were claiming ESA. Table 1 shows the descriptive statistics of the cohort by benefit type for all variables included in the study. There were statistically significant differences ($p<0.001$) between the two client groups across all variables, except for the test of association between SIMD deciles ($p<0.05$). The table also shows jobs starts for each variable, with 5612 JSA clients (62%) and 867 ESA clients (20%) having at least one job start during the two-year programme.

The influence of age and job start

Figure 1 shows how the predicted probability of JSA and ESA clients having a job start at any point during the two-year WP varies according to baseline age and gender. There is a strong negative relationship between age and having a job start for both JSA and ESA clients. Male JSA clients have a higher probability of job start compared to female JSA clients at all ages. However, there is no difference in the probability of a job start for male and female ESA clients.

The influence of age and job start during the two-year WP intervention

Figures 2a and 2b show the predicted probability of job start for all four three-month periods in year 1 of the WP, and the average for all three month periods in the whole of year two of the WP, for 25 and 50 year old JSA (Figure 2a) and ESA clients (Figure 2b), by gender.

For peer review only - http://bmjopen.bmj.com/site/about/guidelines.xhtml

There are marked differences both by age and benefit type when clients are more likely to have a job start throughout the two-year programme. Younger JSA clients have the highest predicted probability of job start in the first three months of their participation in the programme (females 23%, males 28%) which falls off every three months through the programme to 9.6% for females and 11.7% for males in an average three month period in year two of the programme. Older JSA clients have a similar probability of a job start by gender in the first six months of the programme (female 15%, male 18%), which decreases to 5.8% and 6.8% for females and males respectively in an average three month period in year two.

ESA clients, with more health conditions, have a much lower probability of job start compared to JSA clients, particularly in the first three months of the programme (ESA 25 year olds 4%, 50 year olds 2.4%). Apart from a small increase (less than 1%) between four to six months the probability of a job start remains largely unchanged for 25 and 50 year olds ESA clients for the remainder of the programme, with the younger clients having a higher probability.

Factors associated with job start

Unadjusted and multivariable adjusted incident rate ratios (IRR) for factors associated with having a job start for JSA clients are shown in Table 2. Unadjusted analyses showed that female JSA clients were 16% less likely than male JSA clients to have a job start but this association was entirely attenuated when adjusting for all other factors. The length of unemployment before joining the WP was a strong predictor of job start. Clients having educational qualifications under five General Certificate of Secondary Education (GCSEs, or equivalent) or below GSCE level were 17% and

For peer review only - http://bmjopen.bmj.com/site/about/guidelines.xhtml

23% respectively less likely to have a job start compared to those with a degree or higher. Unadjusted analyses showed that compared to white British clients, other clients were 12% more likely to have a job start (but when adjusted this association was not significant).

In terms of clients' health, disclosing one health condition was associated with a 12% decrease, two health conditions a 24% decrease and three or more health conditions a 45% decrease in the likelihood of having a job start compared to disclosing no health conditions. Having health concerns which the client believed would affect their ability to work versus having no health concerns, was associated with a 36% decrease in the likelihood of having a job start. Client perception of job start was a strong predictor of job start.

In terms of personal circumstances having caring responsibilities for anyone other than children and living in insecure housing (which included temporary housing and homelessness) were associated with a decreased likelihood of job start. Although unadjusted analyses showed significant associations with parental status, when adjusted all associations were lost. In terms of external factors JSA clients living in SIMD decile 1 were less likely to have a job start, however the association was lost in the adjusted model. JSA clients living in large urban areas were more likely to have a job start, perhaps reflecting the greater number and types of job opportunities.

Unadjusted and multivariable adjusted IRR for factors associated with having a job start for ESA clients are shown in Table 3. Females were as likely as male clients to have a job start. Increasing length of unemployment was significantly associated with

For peer review only - http://bmjopen.bmj.com/site/about/guidelines.xhtml

a lower probability of having a job start. Whilst there was a relationship between education and RTW in the unadjusted model, this disappeared in the fully adjusted model. Non-white British clients were less likely to have a job start.

For ESA clients, disclosing health conditions was associated with a 16% decrease (two health conditions), 32% decrease (three health conditions), 51% decrease (four health conditions) and 49% decrease (five or more health conditions) in the likelihood of having a job start compared to disclosing zero or one health conditions. Having health concerns which the client believed would affect their ability to work versus having no health concerns, was associated with a 34% decrease in the likelihood of having a job start. Clients' perceptions of job start were a strong predictor of job start. Compared to those clients who thought they would start work within 1 month, clients who thought they would start work in more than 4 months were significantly associated with a reduction in job start in the adjusted model. In terms of personal circumstances only parental status was significantly associated with job start, and those with no children less likely to have a job start. In the unadjusted model those clients living in deciles 2 to 10 were more likely to have a job start than clients living in decile 1 (most deprived) but this association was lost after adjustment in the full model. Urban-rural location showed no association for ESA clients.

For peer review only

For peer review only - http://bmjopen.bmj.com/site/about/guidelines.xhtml

DISCUSSION

This study sought to investigate the role of age, health and other factors associated with RTW among two client groups engaging with the two-year WP. There is a strong continuous negative relationship between age and having a job start for both JSA and ESA clients, with no clear evidence for a specific age at which RTW becomes much less likely. For all JSA clients, the probability of a job start is highest in the first three months from joining the programme, decreasing progressively throughout rest of the programme. For ESA clients the probability of a job start changes little throughout the WP. We further identified a range of factors which were associated with JSA and ESA clients having a job start, including some that have not been explored in previous RTW programmes. Our findings discussed below reveal some benefit type similarities as well as some interesting differences with the literature.

Reducing the disability employment gap and enabling more older people to work for longer are key policy challenges.[12 28] This study is therefore particularly important as it extends our understanding of the factors, including age and health as well as certain socio-economic factors, which are associated with RTW and by investigating the JSA and ESA clients separately we have detected further differences which other studies were not able to discern.[8 30] The unique and rich SOPIE cohort also benefits from a two-year follow up on all clients. While several studies have reviewed RTW in welfare-to-work initiatives, these have been limited in several ways, for example they examine fewer explanatory variables and discrete age categories.[8 37 38] Thus, the size of the SOPIE cohort and the range of the variables collected are considerable strengths. While the availability of such rich data on RWT is a major strength of this study, there are also some limitations to the data. The research team

For peer review only - http://bmjopen.bmj.com/site/about/guidelines.xhtml

only had access to the variables routinely collected and could not specify the data collection. Apart from the employment outcomes the baseline data were generally collected when a client was first referred to the programme and data on external factors such as job opportunities is limited. While this study is limited to clients in Scotland the results are generalizable to the rest of the UK.

In terms of the first research question, older age has been shown to be associated with a lower likelihood of RTW, consistent with many other studies.[8 37-41] However, Figure 2a and b suggest that while in the first 9 months younger JSA clients have a higher likelihood of RTW, after that period the rates differ little by age. For ESA clients the age differentials remain for the full two years of the programme. For research question 2, the highest predicted probability of job start is in the first three months of the programme for JSA clients. These clients could simply be the most employable clients (e.g. in terms of skills, experience etc.) and so be an example of 'creaming' (where the support provider prioritises unemployed claimants with fewer barriers to work and who are therefore felt to be easier and more likely to move into paid work) as suggested in other studies.[42 43] In contrast although much lower than JSA clients, the highest predicted probability of job start for the ESA clients (both young and old) was between 4 and 6 months. This is unsurprising given the ESA clients had been unemployed for much longer than JSA clients, had more health conditions (Table 1) and may require more support or time for the modification of health barriers or their perceived redundancy of skills. Both client groups have a decrease in RTW probability after 6 months indicating that increased support or a change in the type of support (including perhaps a new approach) may be required as time in the programme increases or they may be so far from the labour market that

For peer review only - http://bmjopen.bmj.com/site/about/guidelines.xhtml

they are seen by employers as being virually unemployable in current labour market conditions.[44]

Research question 3 asked what other factors are associated with RTW for JSA and ESA clients. In addition to age, the factors we investigated were drawn from a broad framework of employability covering three main interrelated components, or sets of factors, that influence a person's employability: individual factors, personal circumstances and external factors.[45 46] Although previous studies have shown gender differences in the likelihood of RTW,[8 37-39 47] the gender difference that was observed for JSA clients is lost when all other factors were included in the adjusted model. Those with poorer employment records (especially longer periods of unemployment) on entering the WP were less likely to have a job start and this confirms much prior evidence of having recent work experience prior to claim/entry to programme for RTW.[8 37 38] Our results further confirm earlier evidence that higher qualifications are important in influencing RTW,[38 41] but interestingly not for ESA clients. Whilst there was a relationship between educational qualifications and RTW in the unadjusted model, this disappeared in the fully adjusted model. Further investigation showed this to be due to the relationship between RTW and both length of unemployment and the client's perception of when they saw themselves starting a new job. Ethnicity was important for ESA clients (at the 5% significance level, although not significant for JSA clients) suggesting clients other than white British may require more an enhanced programme.

Health is a major obstacle to the re-employment of benefit claimants.[7 8 37 38] With increasing age the prevalence of long term conditions and disability also increases.[48

For peer review only - http://bmjopen.bmj.com/site/about/guidelines.xhtml

49] Interestingly we found that 29% of the JSA clients disclosed at least one health condition (50% of the over 50 JSA clients) which would suggest that health is still a potential barrier to RTW for JSA clients and should not be ignored. As with other studies, disclosing a health condition (and the novel finding of increasing number) was significantly associated with the decreased likelihood of a job start.[8 39 40 50] Further, health beliefs were important for both client groups confirming the impact of psychosocial factors. Client perception of their likely job start was important for both client groups and negative expectations may reduce resilience and lead to self-fulfilling outcomes of lower RTW. This is consistent with other studies that have shown that clients' own assessment of their ability to RTW was a strong predictor.[51-53]

Personal circumstances included a range related to individuals' social and household circumstances. These may affect the ability, willingness or social pressure for someone to take up an employment opportunity.[54] For JSA clients, childcare had relatively few effects. Hence the, usually gendered, effects of having dependent children seem limited. However for ESA clients having parental responsibilities actually increased the likelihood of job start which may be due to psychosocial factors or a reflection of their level of disability. For non-child caring responsibilities there was a significant impact for JSA clients but the numbers were small (only 4.8% of JSA clients). Housing status was important for JSA clients, although in the adjusted analyses it would appear that it was the clients living in insecure housing that was driving much of this overall negative association. For those in temporary or sheltered housing it is difficult to find a job as some employers may prefer more fixed abode or

For peer review only - http://bmjopen.bmj.com/site/about/guidelines.xhtml

it may be difficult for clients to actually apply of a job if they have no permanent address.[55]

In terms of external factors, as with a WP evaluation,[8] the unadjusted results show that while areas of greatest deprivation were associated with lower RTW, this association was lost in the full models for both client groups. Those JSA clients living in areas other than large urban areas were less likely to have a job start. Other research suggests that the reduction in those claiming benefits is more closely related to the state of the labour market rather than policies or initiatives for RTW,[56] so it was not surprising that JSA clients living in large urban areas were more likely to have a job start, perhaps due to higher levels of labour demand and job opportunities. Further explanations for this could relate to a lack of peer support or role models perhaps and difficulties of transport.[57]

It is widely recognised that being employed can improve a person's health and wellbeing and help reduce health inequalities.[2 5] The key findings of this study have important implications for policy makers. While the disability employment gap has been recognised in Government policy,[29] there is no evidence that new programmes will reduce barriers to the employment of ageing workers. While they will provide specialised support for those unemployed for over two years our findings would suggest much earlier intervention is needed. This is also supported by evidence that the longer an individual is absent from work, the less likely it is that they will return, and early intervention for those off work sick has been shown to be effective.[58 59] Future programmes for those with health conditions or disabilities will be voluntary, and therefore may not engage individuals who because of their unemployment are

For peer review only - http://bmjopen.bmj.com/site/about/guidelines.xhtml

more likely to have low mood, have an inappropriately pessimistic outlook, be socially isolated and reluctant to access support which needs to be based on the biopsychosocial model.[60 61] Perceptions about ability to work are important, but these may have been influenced by health professionals or other advisors with little knowledge of occupational health, workplaces or access to vocational rehabilitation expertise.[58] This study clearly shows that for the individual there is an inverse relationship between job start and the number of health conditions highlighting the need for health care providers to include vocational rehabilitation as part of treatment pathways.[62] Linked to improving workability are education and retraining of ageing workers with medical conditions who may be unfit for their usual role and be disadvantaged because of poor IT skills.[15] At present in the UK much of the educational focus has been on the young unemployed,[63] and new programmes need to include training to update and develop new skills for older workers.

While it generally accepted that most work is beneficial to health,[5] the potential health impacts of engaging with the WP requires further evaluation and linkage of this cohort to National Health Service (NHS) Scotland Information Services Division (ISD) health data is planned. More nuanced estimates of contextual factors such as personal circumstances (including the influence of others in the household) and external factors (such as types of local labour demand and transport provision), would be useful in refining their influence and importance. Future research also needs to evaluate the longer term vocational outcomes of RTW programmes and whether expected health benefits of RTW are realised by these programmes, particularly when distinguishing the types of jobs people enter and the increase in job precariousness and insecurity.

For peer review only - http://bmjopen.bmj.com/site/about/guidelines.xhtml

CONCLUSION

Age, health and a variety of socio-economic factors, play an important role in influencing RTW for unemployed people and for people who have an illness, health condition or disability that makes it difficult to RTW. The results from this study will help inform interventions focussing on addressing age-specific, health and biopsychosocial barriers for future RTW programmes with the aim of improving employment outcomes, so that individuals, society, employers and the economy can benefit from extending working lives.

For peer review only

For peer review only - http://bmjopen.bmj.com/site/about/guidelines.xhtml

Author Statement

EBM, JB, SVK, AHL, RWM and JF had the original idea and developed the overall research design. JB conducted the statistical analysis with significant advice from SVK, AHL, RWM and JF. JB wrote the first draft. All authors subsequently contributed and commented to the manuscript and approved the final version.

Funding

This project (and researcher JB) is funded by the MRC as part of MRC LLHW Extending Working Lives Partnership Awards (MR/L006367/1). AHL and SVK are funded by the MRC (MC_UU_12017/13 and MC_UU_12017/15) and the Scottish Government Chief Scientist Office (SPHSU13 and SPHSU15). SVK is supported by a Fellowship from the Scottish Government Chief Scientist Office (SCAF/15/02). JF is funded by the MRC (MR/K023209/1) and Scottish Government Chief Scientist Office (SCPHRP), as well as the Usher Institute of Population Health Sciences and Informatics at the University of Edinburgh.

Acknowledgments

The authors would like to acknowledge the help of Paul de Pellette, Luke Jeavons, Cem Zobu at Ingeus, Oarabile Molaodi, Daniel Mackay at the University of Glasgow and the DWP.

Ethics

Ethical approval for this project was received from the University of Glasgow College of Social Sciences Research Ethics Committee (400140186).

For peer review only - http://bmjopen.bmj.com/site/about/guidelines.xhtml

Patient consent

Not required.

Competing interests

None declared.

Data sharing statement

Ingeus acts as data processor on behalf of the data controller, the DWP. Ingeus is able to share data with the University of Glasgow (UoG) as detailed in the MRC Collaboration Agreement and the Research Data Licence between UoG and DWP.

For peer review only

For peer review only - http://bmjopen.bmj.com/site/about/guidelines.xhtml

Figure 1 Predicted probability of JSA and ESA clients having a job start by age during the two-year WP

Figure 2a Step graph showing predicted probability of 25 & 50 year old female and male *JSA* clients having a job start in a 3 month period

Figure 2b Step graph showing predicted probability of 25 & 50 year old female and male *ESA* clients having a job start in a 3 months period

For peer review only

For peer review only - http://bmjopen.bmj.com/site/about/guidelines.xhtml

Table 1 Descriptive characteristics and job start of the SOPIE cohort by benefit type

	JSA clients		ESA clients	
	**No. of clients (% of total JSA clients)**	**No. of clients with job start (% of each category with job start)**	**No. of clients (% of total ESA clients)**	**No. of clients with job start (% of each category with job start)**
**Benefit type**	8996	5612 (62.4%)	4322	867 (20.1%)
**Individual factors**				
**Age**				
Under 50	7590 (84.4%)	4919 (64.8%)	3000 (69.4%)	685 (22.8%)
Over 50	1406 (15.6%)	693 (49.3%)	1322 (30.6%)	182 (13.8%)
**Gender**				
Male	5799 (64.5%)	3754 (64.7%)	2260 (52.3%)	450 (19.9%)
Female	3197 (35.5%)	1858 (58.1%)	2062 (47.7%)	417 (20.2%)
**Length of prior unemployment**				
0-6 months	638 (7.1%)	476 (74.6%)	128 (3.0%)	73 (57.0%)
7-12 months	2034 (22.6%)	1498 (73.7%)	264 (6.1%)	118 (44.7%)
1-2 years	3510 (39.0%)	2416 (68.3%)	733 (17.0%)	261 (35.6%)
3-5 years	1072 (11.9%)	571 (53.3%)	802 (18.6%)	173 (21.6%)
6-10 years	886 (9.9%)	399 (45.0%)	885 (20.5%)	119 (13.5%)
11 years+	856 (9.5%)	252 (29.4%)	1510 (34.9%)	123 (8.2%)
**Highest qualification**				
Degree or higher	580 (6.5%)	436 (75.2%)	165 (3.8%)	64 (38.8%)
A levels/NVQ Level 3 and equivalent	1443 (16.0%)	1006 (69.7%)	480 (11.1%)	149 (31.0%)
5 or more GCSEs Grades A*-C and equivalent	1564 (17.4%)	1094 (70.0%)	468 (10.8%)	134 (28.6%)
Under 5 GCSEs A*-C and equivalent	2145 (23.8%)	1317 (61.4%)	975 (22.6%)	185 (19.0%)
Below GSCE level	3264 (36.3%)	1759 (53.9%)	2234 (51.7%)	335 (15.0%)
**Ethnicity**				

For peer review only

For peer review only - http://bmjopen.bmj.com/site/about/guidelines.xhtml

White British	7950 (88.4%)	4906 (61.7%)	4062 (94.0%)	813 (20.0%)
Other	1046 (11.4%)	706 (67.5%)	260 (6.0%)	54 (20.8%)
**Have health concerns which believe will affect ability to work**				
No	7247 (80.6%)	4984 (68.8%)	255 (5.9%)	126 (49.4%)
Yes	1749 (19.4%)	628 (35.9%)	4067 (94.1%)	741 (18.2%)
**Number of health conditions disclosed**				
0	6365 (70.8%)	4399 (69.1%)		
0 and 1			1290 (29.8%)	381 (29.5%)
1	1727(19.2%)	905 (52.4%)		
2	608 (6.8%)	239 (39.3%)	1396 (32.3%)	296 (21.2%)
3			896 (20.7%)	123 (13.7%)
3 or more	296 (3.3%)	69 (23.3%)		
4			425 (9.8%)	40 (9.4%)
5 or more			315 (7.3%)	27 (8.6%)
**Client perception of job start - When do you see yourself starting work?**				
Within 1 month	2188 (24.3%)	1646 (75.2%)	92 (2.1%)	56 (60.9%)
2-3 months	3268 (36.3%)	2258 (69.1%)	248 (5.7%)	142 (57.3%)
4-6 months	875 (9.7%)	486 (55.5%)	262 (6.1%)	117 (44.7%)
>6 months	397 (4.4%)	115 (29.0%)	1302 (30.1%)	140 (10.8%)
Don't know	2268 (25.2%)	1107 (48.8%)	2418 (56.0%)	412 (17.0%)
**Personal circumstances**				
**Caring responsibility for anyone other than children**				
No	8561 (95.2%)	5398 (63.0%)	4046 (93.6%)	822 (20.3%)
Yes	435 (4.8%)	214 (49.2%)	273 (6.4%)	45 (16.3%)
**Housing**				
Homeowner	401 (4.5%)	261 (65.1%)	323 (7.5%)	95 (29.4%)
Living with family	3049 (33.9%)	2155 (69.4%)	609 (14.1%)	146 (24.0%)

For peer review only - http://bmjopen.bmj.com/site/about/guidelines.xhtml

Rented private	1304 (14.5%)	850 (65.2%)	563 (13.0%)	126 (22.4%)
Rented social	3853 (42.8%)	2212 (57.4%)	2655 (61.4%)	469 (17.7%)
Insecure	389 (4.3%)	174 (44.7%)	172 (4.0%)	31 (18.0%)
**Parental status**				
No children	4892 (54.4%)	3218 (65.8%)	1693 (39.2%)	329 (19.4%)
Children, two parent family	622 (6.9%)	406 (65.3%)	288 (6.7%)	89 (30.9%)
Children, shared custody/ not living with you	1254 (13.9%)	735 (58.6%)	711 (16.5%)	145 (20.4%)
Children, lone parent family	1369 (15.2%)	800 (58.4%)	597 (13.8%)	135 (22.6%)
Children, adults living at home/ adults not living at home	859 (9.6%)	453 (52.7)	1033 (23.9%)	169 (16.4%)
**External factors**				
**SIMD deciles**				
1 (most deprived)	2888 (32.1%)	1696 (58.7%)	1497 (34.6%)	229 (15.3%)
2	1891 (21.0%)	1203 (63.6%)	865 (20.0%)	182 (21.0%)
3	1211 (13.5%)	753 (62.2%)	604 (14.0%)	138 (22.9%)
4	864 (9.6%)	549 (63.6%)	409 (9.5%)	81 (19.8%)
5	685 (7.6%)	410 (59.9%)	311 (7.2%)	77 (24.8%)
6	466 (5.2%)	297 (63.7%)	239 (5.5%)	53 (22.2%)
7	331 (3.7%)	238 (71.9%)	123 (2.9%)	31 (25.2%)
8	272 (3.0%)	181 (66.5%)	103 (2.4%)	26 (25.2%)
9	234 (2.6%)	168 (71.8%)	111 (2.6%)	36 (32.4%)
10 (least deprived)	154 (1.7%)	117 (76.0%)	60 (1.4%)	14 (23.3%)
**8-fold urban rural classification**				
1 Large Urban Areas	4573 (50.8%)	2891 (63.2%)	2578 (59.7%)	467 (18.1%)
2 Other Urban Areas	3176 (35.3%)	1962 (61.8%)	1237 (28.6%)	275 (22.2%)
3 Accessible small Towns	623 (6.9%)	395 (63.4%)	232 (5.4%)	57 (24.6%)
4 Remote Small Towns	106 (1.2%)	55 (51.9%)	40 (0.9%)	10 (25.0%)
5 Very Remote Small Towns	67 (0.7%)	32 (47.8%)	29 (0.7%)	10 (34.5%)
6 Accessible Rural	333 (3.7%)	211 (63.4%)	151 (3.5%)	38 (25.1%)

For peer review only - http://bmjopen.bmj.com/site/about/guidelines.xhtml

7 Remote Rural	80 (0.9%)	47 (58.8%)	34 (0.8%)	6 (17.7%)
8 Very Remote Rural	38 (0.4%)	19 (50.0%)	21 (0.5%)	4 (19.1%)

Test of association on all variables and benefit type, p-value< 0.001, except for test of association on SIMD deciles and benefit type, p-value<0.05

For peer review only

For peer review only - http://bmjopen.bmj.com/site/about/guidelines.xhtml

Table 2 Factors associated with job start for *JSA clients*

Variable	Unadjusted IRR (95% CI)*	p-value	Adjusted IRR (95% CI)#	p-value
**Individual factors**				
**Gender**		p<0.001		p=0.425
Male	1.00		1.00	
Female	0.84 (0.80, 0.89)		0.95 (0.89, 1.01)	
**Length of prior unemployment**		p<0.001		p<0.001
0-6 months	1.00		1.00	
7-12 months	0.95 (0.85, 1.05)		0.98 (0.88, 1.08)	
1-2 years	0.80 (0.72, 0.88)		0.84 (0.76, 0.93)	
3-5 years	0.51 (0.45, 0.58)		0.63 (0.56, 0.72)	
6-10 years	0.39 (0.34, 0.44)		0.51 (0.44, 0.58)	
11 years+	0.26 (0.21, 0.28)		0.38 (0.33, 0.45)	
**Highest qualification**		p<0.001		p<0.01
Degree or higher	1.00		1.00	
A levels/NVQ Level 3 and equivalent	0.83 (0.74, 0.92)		0.95 (0.85, 1.07)	
5 or more GCSEs Grades A*-C and equivalent	0.79 (0.71, 0.89)		0.95 (0.84, 1.06)	
Under 5 GCSEs A*-C and equivalent	0.63 (0.56, 0.70)		0.83 (0.74, 0.94)	
Below GSCE level	0.55 (0.49, 0.61)		0.77 (0.69, 0.86)	
**Ethnicity**		p<0.01		p=0.473
White British	1.00		1.00	
Other	1.12 (1.03, 1.21) p<0.01		0.96 (0.89, 1.05)	
**Have health concerns which believe will affect ability to work**		p<0.001		p<0.001
No	1.00		1.00	
Yes	0.41 (0.37, 0.44)		0.64 (0.58, 0.70)	
**Number of health conditions disclosed**		p<0.001		p<0.001
0	1.00		1.00	
1	0.66 (0.61, 0.71)		0.88 (0.82, 0.95)	

For peer review only - http://bmjopen.bmj.com/site/about/guidelines.xhtml

2<br>3 or more	0.46 (0.40, 0.52)<br>0.25 (0.20, 0.32)		0.76 (0.66, 0.88)<br>0.55 (0.43, 0.71)	
**Client perception of job start - When do you see yourself starting work?**		p<0.001		p<0.001
Within 1 month	1.00		1.00	
2-3 months	0.81 (0.76, 0.87)		0.83 (0.78, 0.89)	
4-6 months	0.55 (0.50, 0.61)		0.65 (0.58, 0.72)	
>6 months	0.25 (0.21, 0.31)		0.42 (0.34, 0.51)	
Don't know	0.49 (0.45, 0.53)		0.64 (0.59, 0.69)	
**Personal circumstances**				
**Caring responsibility for anyone other than children**		p<0.001		p<0.001
No	1.00		1.00	
Yes	0.74 (0.64, 0.85)		0.75 (0.66, 0.87)	
**Housing status**		p<0.001		p<0.001
Homeowner	1.00		1.00	
Living with family	0.80 (0.69, 0.92)		0.99 (0.85, 1.14)	
Rented private	0.80 (0.69, 0.92)		1.00 (0.87, 1.15)	
Rented social	0.67 (0.59, 0.76)		0.94 (0.82, 1.07)	
Insecure	0.41 (0.34, 0.50)		0.65 (0.53, 0.79)	
**Parental status**		p<0.01		p=0.485
No children	1.00		1.00	
Children, two parent family	0.98 (0.88, 1.09)		0.97 (0.87, 1.08)	
Children, shared custody/ not living with you	0.83 (0.77, 0.91)		0.93 (0.85, 1.01)	
Children, lone parent family	0.87 (0.79, 0.96)		1.03 (0.94, 1.14)	
Children, adults living at home/ adults not living at home	0.99 (0.88, 1.11)		1.08 (0.96, 1.21)	
**External factors**				
**SIMD deciles**		p<0.001		p=0.162

For peer review only - http://bmjopen.bmj.com/site/about/guidelines.xhtml

1 (most deprived)	1.00		1.00	
2	1.15 (1.07, 1.24)		1.13 (1.05, 1.22)	
3	1.12 (1.03, 1.22)		1.09 (1.00, 1.19)	
4	1.18 (1.07, 1.30)		1.09 (0.99, 1.21)	
5	1.08 (0.97, 1.20)		1.01 (0.91, 1.13)	
6	1.19 (1.05, 1.34)		1.13 (1.00, 1.28)	
7	1.41 (1.23, 1.61)		1.21 (1.05, 1.39)	
8	1.26 (1.08, 1.47)		1.10 (0.94, 1.29)	
9	1.41 (1.21, 1.66)		1.13 (0.96, 1.33)	
10 (least deprived)	1.67 (1.39, 2.02)		1.28 (1.06, 1.56)	
**8-fold urban rural classification**		p<0.05		p<0.05
1 Large Urban Areas	1.00		1.00	
2 Other Urban Areas	0.96 (0.90, 1.02)		0.93 (0.88, 0.99)	
3 Accessible small Towns	1.00 (0.90, 1.11)		1.00 (0.89, 1.11)	
4 Remote Small Towns	0.68 (0.52, 0.89)		0.75 (0.58, 0.99)	
5 Very Remote Small Towns	0.64 (0.45, 0.92)		0.67 (0.47, 0.95)	
6 Accessible Rural	0.99 (0.86, 1.14)		0.96 (0.83, 1.11)	
7 Remote Rural	0.84 (0.63, 1.11)		0.78 (0.59, 1.05)	
8 Very Remote Rural	0.78 (0.50, 1.23)		0.66 (0.42, 1.04)	

*Model contained age and gender

Model adjusted for age, gender, length of unemployment, highest qualification, ethnicity, health concerns, number of health conditions, client perception of job start, caring responsibility, housing status, parental status, SIMD deciles and urban rural classification.

Linear trends are shown for length of unemployment, highest qualification, number of health conditions and SIMD deciles. All other variables show overall trend.

For peer review only - http://bmjopen.bmj.com/site/about/guidelines.xhtml

Table 3 Factors associated with job start for *ESA clients*

Variable	Unadjusted IRR (95% CI)*	p-value	Adjusted IRR (95% CI)#	p-value
**Individual factors**				
**Gender**		p=0.632		p=0.135
Male	1.00		1.00	
Female	1.03 (0.90, 1.18)		1.11 (0.96, 1.30)	
**Length of prior unemployment**		p<0.001		p<0.001
0-6 months	1.00		1.00	
7-12 months	0.67 (0.50, 0.90)		0.70 (0.52, 0.95)	
1-2 years	0.49 (0.38, 0.64)		0.56 (0.42, 0.73)	
3-5 years	0.27 (0.20, 0.35)		0.37 (0.28, 0.50)	
6-10 years	0.16 (0.12, 0.21)		0.23 (0.17, 0.32)	
11 years+	0.10 (0.07, 0.13)		0.17 (0.13, 0.24)	
**Highest qualification**		p<0.001		p=0.319
Degree or higher	1.00		1.00	
A levels/NVQ Level 3 and equivalent	0.78 (0.58, 1.05)		1.17 (0.86, 1.59)	
5 or more GCSEs Grades A*-C and equivalent	0.68 (0.51, 0.92)		1.10 (0.80, 1.51)	
Under 5 GCSEs A*-C and equivalent	0.44 (0.33, 0.59)		0.87 (0.64, 1.18)	
Below GSCE level	0.36 (0.27, 0.47)		0.86 (0.64, 1.15)	
**Ethnicity**		p=0.629		p<0.05
White British	1.00		1.00	
Other	1.07 (0.81, 1.41)		0.70 (0.52, 0.93)	
**Have health concerns which believe will affect ability to work**		p<0.000		p<0.000
No	1.00		1.00	
Yes	0.30 (0.25, 0.37)		0.66 (0.53, 0.82) p<0.001	
**Number of health conditions disclosed**		p<0.000		p<0.000

For peer review only - http://bmjopen.bmj.com/site/about/guidelines.xhtml

0+1 (Note 121 clients disclosed 0 HCs)	1.00		1.00	
2	0.68 (0.58, 0.79)		0.84 (0.72, 0.98)	
3	0.44 (0.36, 0.54)		0.68 (0.55, 0.84)	
4	0.30 (0.22, 0.42)		0.49 (0.35, 0.68)	
5 or more	0.28 (0.19, 0.42)		0.51 (0.34, 0.76)	
**Client perception of job start - When do you see yourself starting work?**		p<0.000		p<0.000
Within 1 month	1.00		1.00	
2-3 months	0.81 (0.60, 1.11)		0.96 (0.69, 1.32)	
4-6 months	0.51 (0.37, 0.71)		0.67 (0.48, 0.94)	
>6 months	0.11 (0.08, 0.15)		0.25 (0.18, 0.35)	
Don't know	0.17 (0.13, 0.22)		0.33 (0.24, 0.45)	
**Personal circumstances**				
**Caring responsibility for anyone other than children**		p=0.313		p=0.568
No	1.00		1.00	
Yes	0.86 (0.63, 1.16)		0.87 (0.64, 1.18)	
**Housing status**		p<0.001		p=0.071
Homeowner	1.00		1.00	
Living with family	0.51 (0.38, 0.67)		0.66 (0.49, 0.88)	
Rented private	0.54 (0.41, 0.71)		0.69 (0.52, 0.91)	
Rented social	0.45 (0.36, 0.56)		0.72 (0.57, 0.91)	
Insecure	0.38 (0.25, 0.58)		0.59 (0.38, 0.90)	
**Parental status**		p=0.351		p<0.01
No children	1.00		1.00	
Children, two parent family	1.61 (1.27, 2.04)		1.43 (1.12, 1.84)	
Children, shared custody/ not living with you	1.06 (0.87, 1.29)		1.18 (0.96, 1.44)	
Children, lone parent family	1.12 (0.90, 1.39)		1.24 (0.99, 1.56)	
Children, adults living at home/ adults not	1.13 (0.92, 1.40)		1.28 (1.03, 1.60)	

For peer review only - http://bmjopen.bmj.com/site/about/guidelines.xhtml

living at home				
**External factors**				
**SIMD deciles**		p<0.001		p=0.184
1 (most deprived)	1.00		1.00	
2	1.40 (1.15, 1.70)		1.41 (1.15, 1.72)	
3	1.55 (1.26, 1.92)		1.39 (1.12, 1.73)	
4	1.33 (1.03, 1.72)		1.09 (0.84, 1.41)	
5	1.72 (1.33, 2.22)		1.24 (0.94, 1.62)	
6	1.51 (1.12, 2.03)		0.96 (0.71, 1.31)	
7	1.86 (1.28, 2.71)		1.50 (1.02, 2.21)	
8	1.75 (1.17, 2.63)		1.29 (0.85, 1.95)	
9	2.45 (1.72, 3.49)		1.53 (1.06, 2.22)	
10 (least deprived)	1.59 (0.93, 2.73)		1.00 (0.58, 1.75)	
**8-fold urban rural classification**		p=0.678		p=0.210
1 Large Urban Areas	1.00		1.00	
2 Other Urban Areas	1.22 (1.05, 1.42)		1.00 (0.85, 1.16)	
3 Accessible small Towns	1.39 (1.06, 1.83)		1.08 (0.81, 1.43)	
4 Remote Small Towns	1.40 (0.75, 2.61)		1.13 (0.60, 2.14)	
5 Very Remote Small Towns	1.98 (1.06, 3.71)		2.15 (1.14, 4.07)	
6 Accessible Rural	1.51 (1.08, 2.10)		1.04 (0.74, 1.47)	
7 Remote Rural	0.99 (0.44, 2.21)		0.74 (0.32, 1.67)	
8 Very Remote Rural	1.18 (0.44, 3.17)		0.85 (0.31, 2.30)	

*Model contained age and gender

Model adjusted for age, gender, length of unemployment, highest qualification, ethnicity, health concerns, number of health conditions, client perception of job start, caring responsibility, housing status, parental status, SIMD deciles and urban rural classification.

Linear trends are shown for length of unemployment, highest qualification, number of health conditions and SIMD deciles. All other variables show overall trend.

For peer review only - http://bmjopen.bmj.com/site/about/guidelines.xhtml

For peer review only

References

1. Akinwale B, Lynch K, Wiggins R, et al. Work, permanent sickness and mortality risk: a prospective cohort study of England and Wales, 1971-2006. J Epidemiol Community Health 2011;**65**(9):786-92 doi: 10.1136/jech.2009.099325[published Online First: Epub Date]|.
2. Black C. Working for a healthier tomorrow: Dame Carol Black's Review of the health of Britain's working age population. London, 2008.
3. Katikireddi SV, Leyland AH, McKee M, et al. Patterns of mortality by occupation in the UK, 1991–2011: a comparative analysis of linked census and mortality records. The Lancet Public Health 2017;**2**(11):e501-e12 doi: 10.1016/S2468-2667(17)30193-7[published Online First: Epub Date]|.
4. Katikireddi SV, Niedzwiedz CL, Popham F. Employment status and income as potential mediators of educational inequalities in population mental health. European journal of public health 2016;**26**(5):814-16 doi: 10.1093/eurpub/ckw126[published Online First: Epub Date]|.
5. Waddell G, Burton K. Is work good for your health and wellbeing? The Stationery Office, London, 2006.
6. Bartley M, Sacker A, Clarke P. Employment status, employment conditions, and limiting illness: prospective evidence from the British household panel survey 1991-2001. J Epidemiol Community Health 2004;**58**:501-06
7. Houston D, Lindsay C. Fit for work? Health, employability and challenges for the UK welfare reform agenda. Policy Studies 2010;**31**(2):133-42
8. Meager N, Newton B, Sainsbury R, et al. Work Programme Evaluation : the participant experience report. Department for Work and Pensions, London, 2014.
9. Thomas C, Benzeval M, Stansfeld S. Employment transitions and mental health: an analysis from the British household panel survey. J Epidemiol Community Health 2005;**59**(3):243-49
10. Altmann R. A New Vision for Older Workers: Retain, Retrain, Recruit. Department for Work and Pensions, London, 2015.
11. Green A, de Hoyos M, Barnes S, et al. Literature review on Employability, Inclusion and ICT, Report 1: The Concept of Employability, with a Specific Focus on Young People, Older Workers and Migrants. Luxembourg, 2013.
12. Department for Work and Pensions. Fuller Working Lives Evidence Base - 2017, 2017.
13. Harper S, Walport M. Future of an Ageing Population. London, 2016.
14. Cappellari L, Dorsett R, Haile G. Labour market transitions among the over-50s. Department for Work & Pensions, London, 2005.
15. Ilmarinen J. Aging workers. Occupational and Environmental Medicine 2001;**58**(8):546-52
16. McNair S. How different is the older labour market? Attitudes to work and retirement among older people in Britain. Social Policy & Society 2006;**5**:485-94
17. Riach M, Loretto W. Identity work and the 'unemployed' worker: age, disability and the lived experience of the older unemployed. Work, employment and society 2009;**23**(1):102-19
18. Canduela J, Dutton M, Johnson S, et al. Ageing, skills and participation in work-related training in Britain: assessing the position of older workers. Work Employ Soc 2012;**26**(1):42-60.

For peer review only - http://bmjopen.bmj.com/site/about/guidelines.xhtml

19. Gayen K, McQuaid R, Raeside R. Social networks, age cohorts and employment. International Journal of Sociology and Social Policy 2010;**30**(5/6):219 - 38.
20. Taylor P, Walker A. The Ageing Workforce: Employers' Attitudes Towards the Employment of Older People. Work Employment & Society 1994; **8**(4):569-91
21. Weir G. The economically inactive who look after the family or home. Labour Market Trends 2002;**110**:577-87
22. Fuertes V, Egdell V, R M. Extending working lives: Age management in SMEs. Employee Relations 2013;**35**(3):272-93
23. Ritchie H, Casebourne J, J R. Understanding workless people and communities: A literature review. Department for Work and Pensions, London, 2005.
24. Department for Work and Pensions. The Work Programme,. Department for Work and Pensions, London (http://www.dwp.gov.uk/docs/the-work-programme.pdf), 2012.
25. Morse A. The Work Programme. National Audit Office, 2014.
26. The UK Government. Jobseeker's Allowance. Secondary Jobseeker's Allowance 2017. https://www.gov.uk/jobseekers-allowance/eligibility [19/09/2017].
27. The UK Government. Employment and Support Allowance. Secondary Employment and Support Allowance 2017. https://www.gov.uk/employment-support-allowance [19/09/2017].
28. Department for Work and Pensions, Department of Health. Improving Lives: The Work, Health and Disability Green Paper, 2016.
29. Department for Work and Pensions, Department for Health. Improving Lives: The Future of Work, Health and Disability, 2017.
30. Foster S, Colechin J, Bivand P, et al. Employment support for unemployed older people, 2014.
31. Age UK. Helping 50+ jobseekers back to work:lessons for the Work and Health Programme, 2016.
32. Brown J, Neary J, Katikireddi SV, et al. Protocol for a mixed-methods longitudinal study to identify factors influencing return to work in the over 50s participating in the UK Work Programme: Supporting Older People into Employment (SOPIE). Bmj Open 2015;**5**(12) doi: 10.1136/bmjopen-2015-010525[published Online First: Epub Date]|.
33. The Scottish Government. Scottish Neighbourhood Statistics Guide. Secondary Scottish Neighbourhood Statistics Guide. Available from http://www.gov.scot/Publications/2005/02/20697/52626 [05/10/15].
34. The Scottish Government. Scottish Government Urban Rural Classification. Secondary Scottish Government Urban Rural Classification. http://www.gov.scot/Topics/Statistics/About/Methodology/UrbanRuralClassification [19/09/17].
35. The Scottish Government. Scottish Index of Multiple Deprivation. Secondary Scottish Index of Multiple Deprivation. Available from: http://www.gov.scot/Topics/Statistics/SIMD [11/09/15].
36. Royston P, Ambler G, Sauerbrei W. The use of fractional polynomials to model continuous risk variables in epidemiology. International Journal of Epidemiology 1999;**28**(5):964-74 doi: 10.1093/ije/28.5.964[published Online First: Epub Date]|.
37. Kemp PA, Davidson J. Employability trajectories among new claimants of Incapacity Benefit. Policy Studies 2010;**31**(2):203-21 doi: 10.1080/01442870903429637[published Online First: Epub Date]|.

For peer review only - http://bmjopen.bmj.com/site/about/guidelines.xhtml

38. Sissons P, Barnes H. Getting back to work? Claim trajectories and destinations of Employment and Support Allowance claimants. Journal of Poverty and Social Justice 2013;**21**(3):233-46
39. Cancelliere C, Donovan J, Stochkendahl MJ, et al. Factors affecting return to work after injury or illness: best evidence synthesis of systematic reviews. Chiropractic & manual therapies 2016;**24**(1):32 doi: 10.1186/s12998-016-0113-z[published Online First: Epub Date]|.
40. Ervasti J, Joensuu M, Pentti J, et al. Prognostic factors for return to work after depression-related work disability: A systematic review and meta-analysis. Journal of psychiatric research 2017;**95**:28-36 doi: 10.1016/j.jpsychires.2017.07.024[published Online First: Epub Date]|.
41. Schuring M, Robroek SJW, Otten FWJ, et al. The effect of ill health and socioeconomic status on labor force exit and re-employment: a prospective study with ten years follow-up in the Netherlands. Scand J Work Environ Health 2013;**39**(2):134-43 doi: 10.5271/sjweh.3321[published Online First: Epub Date]|.
42. Carter E, Whitworth A. Creaming and Parking in Quasi-Marketised Welfare-to-Work Schemes: Designed Out Of or Designed In to the UK Work Programme? J Soc Policy 2015;**44**(2):277-96.
43. Rees J, Whitworth A, Carter E. Support for All in the UK Work Programme? Differential Payments, Same Old Problem. Social policy & administration 2014;**48**(2):221-39 doi: 10.1111/spol.12058[published Online First: Epub Date]|.
44. Saint-Paul G. Are the unemployed unemployable? European Economic Review 1996;**40**:1501-19
45. Koen J, Klehe U-C, Van Vianen A. Employability among the long-term unemployed: A futile quest or worth the effort? Journal of Vocational Behavior 2013;**82**:37-48
46. McQuaid RW, Lindsay C. The Concept of Employability. Urban Studies 2005;**42**(2):197-219
47. Beatty C, Fothergill S, Houston D, et al. Bringing Incapacity Benefit numbers down: to what extent do women need a different approach? Policy Studies 2010;**31**(2):143-62
48. Barnett K, Mercer SW, Norbury M, et al. Epidemiology of multimorbidity and implications for health care, research, and medical education: a cross-sectional study. Lancet (London, England) 2012;**380**(9836):37-43 doi: 10.1016/s0140-6736(12)60240-2[published Online First: Epub Date]|.
49. Department of Health. Long Term Conditions Compendium of Information: Third Edition, 2012.
50. Lindsay C, Greve B, Cabras I, et al. Assessing the evidence base in health, employability and the labour market - lessons for activation in the UK. 49 2015;**2**:143-60
51. McQuaid RW, Bond S, Fuertes V. Working for Families evaluation. Scottish Government, Edinburgh, 2009.
52. Nieuwenhuijsen K, Noordik E, van Dijk FJH, et al. Return to Work Perceptions and Actual Return to Work in Workers with Common Mental Disorders. J Occup Rehabil 2013;**23**(2):290-99 doi: 10.1007/s10926-012-9389-6[published Online First: Epub Date]|.

For peer review only - http://bmjopen.bmj.com/site/about/guidelines.xhtml

53. Wilford J, McMahon AD, Peters J, et al. Predicting job loss in those off sick. Occupational medicine (Oxford, England) 2008;**58**(2):99-106 doi: 10.1093/occmed/kqm141[published Online First: Epub Date]|.
54. Nauta A, van Vianen A, van der Heijden B, et al. Understanding the factors that promote employability orientation: The impact of employability culture, career satisfaction, and role breadth self-efficacy. Journal of Occupational and Organizational Psychology 2009;**82**(2):233-51 doi: 10.1348/096317908x320147[published Online First: Epub Date]|.
55. Ahmed A, Granberg M, Lång E. Does having one's own place to live make someone more employable? Applied Economics Letters 2017;**24**(18):1327-30 doi: 10.1080/13504851.2016.1276263[published Online First: Epub Date]|.
56. Webster D, Arnott J, Brown J, et al. Falling Incapacity Benefit claims in a former industrial city: policy impacts or labour market improvement? Policy Studies 2010;**31**(2):163-85.
57. McQuaid RW, Chen T. Commuting times - The role of gender, children and part-time work. Research in Transportation Economics 2012;**34**(1):66-73
58. Black C, Frost D. Health at work - an independent review of sickness absence. Secondary Health at work - an independent review of sickness absence 2011 2011.
59. Brown J, Mackay D, Demou E, et al. The EASY (Early Access to Support for You) sickness absence service: a four-year evaluation of the impact on absenteeism. Scand J Work Environ Health 2015;**41**(2):204-15 doi: 10.5271/sjweh.3480[published Online First: Epub Date]|.
60. Demou E, Gibson I, Macdonald EB. Identification of the factors associated with outcomes in a Condition Management Programme. Bmc Public Health 2012;**12**:10 doi: 10.1186/1471-2458-12-927[published Online First: Epub Date]|.
61. Engel G. The need for a new medical model: a challenge for biomedicine. Science 1977;**196**:129-36
62. Health Works. A review of the Scottish Government's Healthy Working Lives Strategy. Secondary Health Works. A review of the Scottish Government's Healthy Working Lives Strategy 2013 2013.
63. Maguire S. NEET, unemployed, inactive or unknown – why does it matter? Educational Research 2015;**57**(2):121-32 doi: 10.1080/00131881.2015.1030850[published Online First: Epub Date]|.

For peer review only

For peer review only - http://bmjopen.bmj.com/site/about/guidelines.xhtml

For peer review only

For peer review only - http://bmjopen.bmj.com/site/about/guidelines.xhtml

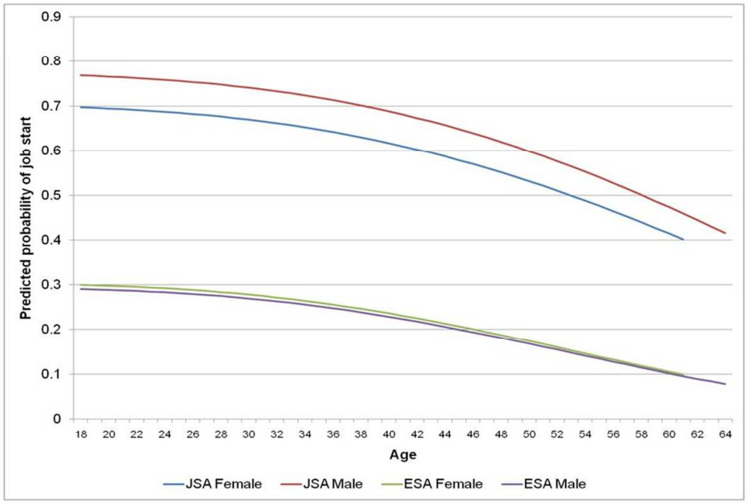

Figure 1 Predicted probability of JSA and ESA clients having a job start by age during the two-year WP

108x60mm (300 x 300 DPI)

For peer review only

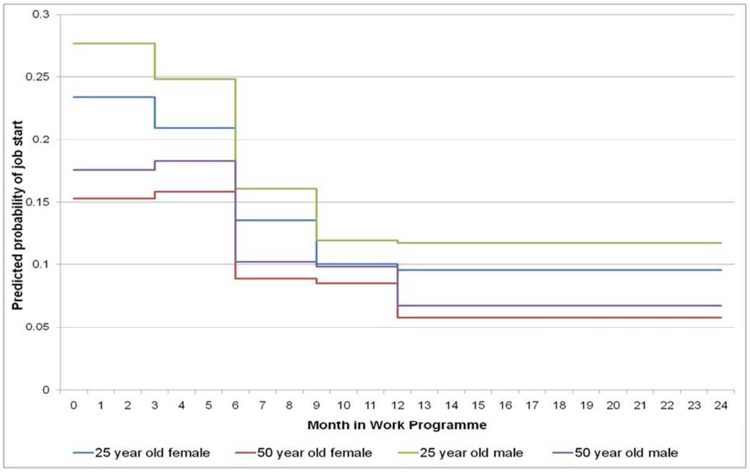

Figure 2a Step graph showing predicted probability of 25 & 50 year old female and male JSA clients having a job start in a 3 month period

108x60mm (300 x 300 DPI)

review only

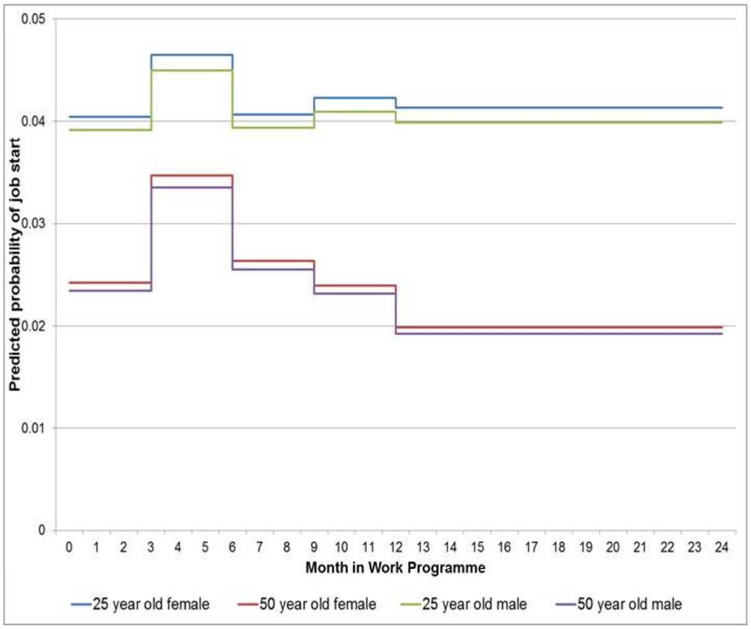

Figure 2b Step graph showing predicted probability of 25 & 50 year old female and male ESA clients having a job start in a 3 months period

81x60mm (300 x 300 DPI)

For peer review only - http://bmjopen.bmj.com/site/about/guidelines.xhtml

STROBE 2007 (v4) Statement—Checklist of items that should be included in reports of *cohort studies*

Section/Topic	Item #	Recommendation	Reported on page #
**Title and abstract**	1	(*a*) Indicate the study's design with a commonly used term in the title or the abstract	1
		(*b*) Provide in the abstract an informative and balanced summary of what was done and what was found	2 & 3
**Introduction**			
Background/rationale	2	Explain the scientific background and rationale for the investigation being reported	5
Objectives	3	State specific objectives, including any prespecified hypotheses	8
**Methods**			
Study design	4	Present key elements of study design early in the paper	10
Setting	5	Describe the setting, locations, and relevant dates, including periods of recruitment, exposure, follow-up, and data collection	10
Participants	6	(*a*) Give the eligibility criteria, and the sources and methods of selection of participants. Describe methods of follow-up	10
		(*b*) For matched studies, give matching criteria and number of exposed and unexposed	N/A
Variables	7	Clearly define all outcomes, exposures, predictors, potential confounders, and effect modifiers. Give diagnostic criteria, if applicable	10 & 11
Data sources/ measurement	8*	For each variable of interest, give sources of data and details of methods of assessment (measurement). Describe comparability of assessment methods if there is more than one group	10 & 11
Bias	9	Describe any efforts to address potential sources of bias	No specific biases expected. The data was based on 100% sample of Ingeus Work Programme clients. Note that Ingeus clients covered 50% of all Scottish WP clients and were allocated randomly to the Ingeus WP by DWP.
Study size	10	Explain how the study size was arrived at	10 and Protocol

For peer review only

			paper (reference 32)
Quantitative variables	11	Explain how quantitative variables were handled in the analyses. If applicable, describe which groupings were chosen and why	10, 11 & 27 (Table 1)
Statistical methods	12	(*a*) Describe all statistical methods, including those used to control for confounding	11 & 12
		(*b*) Describe any methods used to examine subgroups and interactions	11
		(*c*) Explain how missing data were addressed	10. All missing data (including age) was removed at data cleaning stage.
		(*d*) If applicable, explain how loss to follow-up was addressed	N/A
		(*e*) Describe any sensitivity analyses	N/A
**Results**			
Participants	13*	(a) Report numbers of individuals at each stage of study—eg numbers potentially eligible, examined for eligibility, confirmed eligible, included in the study, completing follow-up, and analysed	After data cleaning 13318 clients, all with follow up
		(b) Give reasons for non-participation at each stage	N/A
		(c) Consider use of a flow diagram	We describe the clients we removed on p10 and is straightforward so don't think a flow diagram is needed.
Descriptive data	14*	(a) Give characteristics of study participants (eg demographic, clinical, social) and information on exposures and potential confounders	13 & 27 (Table 1)
		(b) Indicate number of participants with missing data for each variable of interest	The 947 clients with missing data represented 6.6% of the original cohort. Missing age n=693, postcode (and therefore external factors) n=100, < age 18 n=7, length of unemployment not possible n=147

For peer review only

		(c) Summarise follow-up time (eg, average and total amount)	13
Outcome data	15*	Report numbers of outcome events or summary measures over time	13 & 14
Main results	16	(*a*) Give unadjusted estimates and, if applicable, confounder-adjusted estimates and their precision (eg, 95% confidence interval). Make clear which confounders were adjusted for and why they were included	14, 15, 31 (Table 2) , 34 (Table 3)
		(*b*) Report category boundaries when continuous variables were categorized	We followed outline of good practice.[1]We use deciles for SIMD data and continuous data for age (where the dichotomised ages were presented they were based on theory, empirical papers and the results presented earlier in the paper). The ESA v JSA dichotomy reflected the population who were enrolled in these.
		(*c*) If relevant, consider translating estimates of relative risk into absolute risk for a meaningful time period	N/A
Other analyses	17	Report other analyses done—eg analyses of subgroups and interactions, and sensitivity analyses	N/A
**Discussion**			
Key results	18	Summarise key results with reference to study objectives	17
**Limitations**			
Interpretation	20	Give a cautious overall interpretation of results considering objectives, limitations, multiplicity of analyses, results from similar studies, and other relevant evidence	17
Generalisability	21	Discuss the generalisability (external validity) of the study results	18
**Other information**			
Funding	22	Give the source of funding and the role of the funders for the present study and, if applicable, for the original study on which the present article is based	24

For peer review only

*Give information separately for cases and controls in case-control studies and, if applicable, for exposed and unexposed groups in cohort and cross-sectional studies.

Note: An Explanation and Elaboration article discusses each checklist item and gives methodological background and published examples of transparent reporting. The STROBE checklist is best used in conjunction with this article (freely available on the Web sites of PLoS Medicine at http://www.plosmedicine.org/, Annals of Internal Medicine at http://www.annals.org/, and Epidemiology at http://www.epidem.com/). Information on the STROBE Initiative is available at www.strobe-statement.org.

1. Turner EL, Dobson JE, Pocock SJ. Categorisation of continuous risk factors in epidemiological publications: a survey of current practice. Epidemiologic perspectives & innovations : EP+I 2010;**7**:9 doi: 10.1186/1742-5573-7-9[published Online First: Epub Date].

For peer review only

BMJ Open

Age, health and other factors associated with return to work for those engaging with a welfare-to-work initiative: A cohort study of administrative data from the UK's Work Programme

Journal:	*BMJ Open*
Manuscript ID	bmjopen-2018-024938.R1
Article Type:	Research
Date Submitted by the Author:	07-Sep-2018
Complete List of Authors:	Brown, Judith; University of Glasgow, Institute of Health and Wellbeing<br>Katikireddi, Srinivasa; University of Glasgow School of Life Sciences, MRC/CSO Social & Public Health Sciences Unit<br>Leyland, Alastair; MRC/CSO Social and Public Health Sciences Unit, University of Glasgow,<br>McQuaid, Ronald; University of Stirling, Management Work and Organisation<br>Frank, John; University of Edinburgh, The Usher Institute of Population Health Sciences and Informatics, College of Medicine and Veterinary Medicine,<br>Macdonald, Ewan; University of Glasgow, Institute of Health and Wellbeing
**Primary Subject Heading**:	Public health
Secondary Subject Heading:	Rehabilitation medicine
Keywords:	Ageing, Health, Unemployment, Return to work, Vocational rehabilitation

SCHOLARONE™
Manuscripts

Age, health and other factors associated with return to work for those engaging with a welfare-to-work initiative: A cohort study of administrative data from the UK's Work Programme

Judith Brown,[1] Srinivasa Vittal Katikireddi,[2] Alastair H Leyland,[2] Ronald W McQuaid,[3] John Frank,[4] Ewan B Macdonald[1]

[1] Healthy Working Lives Group, Institute of Health and Wellbeing, College of Medical, Veterinary and Life Sciences, University of Glasgow, Glasgow, G12 8RZ

[2] MRC /CSO Social and Public Health Sciences Unit, Institute of Health and Wellbeing, College of Medical, Veterinary and Life Sciences, University of Glasgow, Glasgow, G12 8RZ

[3] Management, Work and Organisation, University of Stirling, Stirling, FK9 4LA

[4] Usher Institute of Population Health Sciences and Informatics, Teviot Hall, University of Edinburgh, Edinburgh, EH8 9AG

Correspondence to: Dr Judith Brown

Judith.Brown@glasgow.ac.uk

Key words: ageing; health; unemployment; return to work; vocational rehabilitation

Word Count: 5104

For peer review only

For peer review only - http://bmjopen.bmj.com/site/about/guidelines.xhtml

ABSTRACT

Objectives

To investigate the role of individual factors (including age, health and personal circumstances) and external factors associated with clients having a job start while engaging with the Work Programme and variations by benefit type.

Setting

The UK Government's main return to work initiative (The Work Programme) in Scotland.

Design

Piecewise Poisson regression to calculate incident rate ratios (IRR) using administrative data from 2013 to 2016 to identify factors associated with job start.

Participants

4322 Employment Support Allowance (ESA) clients not in work due to poor health and 8996 Jobseeker's Allowance (JSA) clients, aged 18–64 years, referred to the Work Programme between April 2013 and July 2014.

Main outcome measures

Starting a job and the time to first job start after entering the Work Programme.

Results

JSA clients (62%) were more likely to return to work (RTW) than ESA clients (20%). There is a strong negative relationship between age and the predicted probability of having a job start during the two-year engagement with the programme for both JSA and ESA clients. JSA clients were most likely to RTW in the first three months, while for ESA clients the predicted probability of having a first job start was fairly constant over the two years. Health, including the number of health conditions, length of

For peer review only

unemployment, client perception of job start and other individual factors were associated with job starts for both groups.

Conclusions

Age plays an important role in influencing RTW, however important potentially modifiable factors include the length of unemployment, the management of multimorbidity, and the individual's perception of the likelihood of job start. Future welfare-to-work programmes may be improved by providing age-specific interventions which focus on health and biopsychosocial factors to enable more people to realise the potential health benefits of RTW.

For peer review only

For peer review only - http://bmjopen.bmj.com/site/about/guidelines.xhtml

Article Summary

Strength and limitations of study

- This is the first study to explore the role of individual age, health and other factors in returning to work, using large-scale administrative data collected over a participant's two years participation in the Work Programme.
- The use of administrative data (rather than survey data as in previous evaluations) limited loss in follow up, however the analysis relies on routine operational data with limited health diagnostic and severity information available.
- JSA and ESA clients were analysed separately showing important differences in return to work between the two client groups (by definition those with and without an illness, health condition or disability that makes it difficult to work, although many JSA clients disclosed health conditions).
- The number of (self-disclosed) health conditions for JSA and ESA provides new insights.
- This study had modelled age as a continuous variable (1406 JSA clients and 1322 ESA clients aged 50 and over) to better understand the relationship between age and RTW rather than the single age category (50+) of other studies.

For peer review only - http://bmjopen.bmj.com/site/about/guidelines.xhtml

INTRODUCTION

Labour market participation is an important determinant of health and health inequalities, with efforts to increase paid employment thought by policymakers to improve health.[1-5] However, health status also inhibits returning to work from being unemployed, especially for those with multiple health problems.[6-9] In addition, age is closely connected to both health status and other difficulties in returning to work, with employment rates for the working age population declining sharply from over 80% of 50 year olds in the UK, to around 60% of 60 year-olds with a steeper decline at older ages.[10] This paper uses a unique database to analyse the likelihood of unemployed people on the government's Work Programme (WP) returning to work. Specifically it considers the role of age when comparing those with an illness, health condition or disability that makes it difficult to work (those receiving the Employment and Support Allowance) and others on the programme (those receiving Jobseeker's Allowance).

Due to the ageing population, older workers increasingly make up a greater proportion of the workforce in most economically developed countries.[11] Thus recruiting and retaining older workers, and encouraging and enabling more people to work for longer, is a policy priority for governments and employers.[11 12] For instance, from 2014 to 2024 the UK will have 200,000 fewer people aged 16 to 49 and 3.2 million more people aged 50 to state pension age, but the latter with a lower average employment rate.[13]

Older workers face significant barriers in the labour market and are less likely to regain employment after job loss and are at increased risk of chronic health conditions

For peer review only - http://bmjopen.bmj.com/site/about/guidelines.xhtml

which contribute to job loss and may make re-employment difficult.[14-17] In addition, this age group may encounter barriers and factors that interact with their health including direct and indirect age discrimination,[18-20] skills gaps (especially in IT),[19] caring responsibilities, e.g. for grandchildren or elderly parents,[21] and the lack of flexible working opportunities.[22] Some individuals may experience multiple interacting or overlapping disadvantages which may be difficult to resolve in isolation.[8 23] For older workers (in this study refers to those aged 50 to 64) there are added difficulties of separating the impact of biological ageing from the impacts of unemployment and health selection effects (for example, ill health leading to early voluntary retirement).[5]

The WP was the UK Government's flagship welfare-to-work initiative to help those more detached from the labour market to enter employment and reduce the time people spent on benefits. The design of the WP has parallels with other Organisation for Economic Cooperation and Development (OECD) countries active labour market policies for those on welfare or unemployed, in terms of moves towards delivery of general and specialist employment services through networks of private and not-for-profit organisations, usually through employment outcomes based performance contracts, with a variety of forms of procurement.[24 25] In addition to the WP, outsourcing included services for the disabled in countries such as Australia and the Netherlands (mainly to not-for-profit or private organisations), Sweden, Denmark and USA.[26-30]

The WP was launched throughout Great Britain in June 2011 as part of a sweeping programme of welfare reforms with final referrals in March 2017.[31] It required

For peer review only - http://bmjopen.bmj.com/site/about/guidelines.xhtml

more people to either seek work or to undertake some form of work-related activity as a condition of receiving benefit.[32] The two-year programme was delivered by a range of private, public and voluntary sector organisations across eighteen regions. The Department for Work and Pensions (DWP) paid two or more prime contractors to provide directly or indirectly (through sub-contractors) support to unemployed job seekers in each region (delivered as a 'black box' approach where much of the control over services provided was wielded by the contractor). One innovatory feature was that payments were by results with most payments being after a participant had sustained employment (although not necessary with the same employer) for a minimum time, rather than payments being mainly for job seekers participating or entering employment.

The unemployed including those out of work due to health reasons were required to participate in the WP, and others were able to volunteer to use the services depending on their circumstances. The WP supported two main groups of benefit claimants: Jobseeker's Allowance (JSA) clients and Employment and Support Allowance (ESA) clients. JSA is a benefit for people who are unemployed but capable of work, usually paid to unemployed people if all of the following apply: they are aged 18 to State Pension age, not in full-time education, living in Great Britain, available for work, actively seeking work and work on average less than 16 hours per week.[33] Those aged 18-24 years were a priority group and had to have been unemployed a shorter period (usually 6 months) before entering the WP than most older groups. ESA is a benefit for people who have an illness, health condition or disability that makes it difficult to work and requires participants to undergo a Work Capability Assessment.[34] Claimants may get ESA if their illness or disability affects their

For peer review only - http://bmjopen.bmj.com/site/about/guidelines.xhtml

ability to work and if they are: under State Pension age, not getting Statutory Sick Pay or Statutory Maternity Pay and have not gone back to work, and are not getting Jobseeker's Allowance. ESA clients are not separated by age and often people become disabled or experience worsening health, and so join ESA, later in life. So generally ESA clients in our study are older than JSA clients.

One of the most extensive reports from the national WP evaluation to investigate factors influencing return to work (RTW) is a telephone survey of 4700 clients and a follow up survey of 1800 of the same clients.[8] It found that after two years on the programme, 67% of people were still not in work, and were more likely to be male, older than 55, have health conditions, few qualifications, and no recent work experience. Because having a health condition or disability was shown to be an important factor we believe there is a need to investigate ESA clients separately since people with long-term health conditions and disabilities experience disproportionately lower employment rates. Data from 2015 show a significant gap in the employment rates of disabled (48%) and non-disabled people (80%),[35] and while the UK Government is committed to seeing one million more people in work over the next ten years,[36] if there is to be any chance of achieving this, then there must be a focus on improving outcomes for the over 50s and for those with disabilities in these new schemes. Furthermore, the evaluation investigated clients by age categories (18-24, 25-34, 35-44, 45-54, 55-59 & 60+) and for some analyses grouped clients aged 50+ into one group. The National Audit report of the WP did investigate JSA clients (termed easier-to-help) and ESA clients (termed harder-to-help) separately but they only reported on performance in getting people into work and not on the factors associated with RTW.[32] AgeUK reports divided over 50s into three age categories

For peer review only

(50-54, 55-59, 60+) and suggested that low job performance is not caused by a higher incidence of disability or health conditions but rather age itself was the main barrier to work.[37 38]

In order to better understand all clients aged 50 and above we investigated individual factors, personal circumstances and external factors associated with RTW of JSA and ESA clients separately, treating age as a continuous variable. The aim of this study was to answer the following research questions:

1. What is the relationship between age and returning to work for JSA and ESA clients engaged with the WP?

2. How does the likelihood of returning to work change over the period of their participation in the WP for JSA and ESA clients?

3. What other factors, including health, are associated with RTW for JSA and ESA clients?

For peer review only

For peer review only - http://bmjopen.bmj.com/site/about/guidelines.xhtml

METHODS

Description of cohort

'Supporting Older people into Employment' (SOPIE) is a mixed methods longitudinal study involving a collaboration between academics, a major WP provider (Ingeus) and the UK DWP. Full details on the study, including sample size, can be found in the protocol paper.[39] The study population was all clients who entered the Ingeus WP in Scotland between the 1st April 2013 and the 31st July 2014 (14265 clients). After data cleaning the SOPIE cohort totalled 13318 clients. The 947 clients were removed from the study population as they: had significant missing baseline data including age (n=693); it was not able to generate datazone from postcode (n=100); were aged 16 to 17 (n=7); had lengths of unemployment which were not possible, for example a 20 year old with 10 years of unemployment history (n=147). The cohort was followed up longitudinally for the two years they engaged with Ingeus on the WP.

Variables

After referral to the WP clients completed a baseline face-to-face assessment with an employment advisor. The individual factors collected in the baseline assessment and used in this study were: age, gender, length of unemployment prior to the WP, highest qualification, ethnicity, whether the client had health concerns which they believed would affect their ability to work, number of health conditions disclosed to advisor, client perception of their likelihood of starting a job, and personal circumstances (caring responsibility other than children, housing status, parental status). Ethnicity was recoded as white British and all other due to sample size. The number of health conditions disclosed by clients were categorised into 0, 1, 2 and 3 or more for JSA clients and 0/1, 2, 3, 4 and 5 or more for ESA clients. As expected ESA clients

For peer review only - http://bmjopen.bmj.com/site/about/guidelines.xhtml

disclosed a greater number of health conditions. 121 (2.8%) ESA clients disclosed no health conditions. This may have been a coding error or the client may have decided not to disclose their health condition to their advisor hence we coded 0 and 1 disclosed health conditions together (see Table 1).

Analyses of external factors were conducted using datazones. The 6976 datazones in Scotland have populations of between 500 and 1,000 household residents,[40] and Ingeus determined the datazone from the client's postcode. For each client the research team added the 2016 Scottish Index of Multiple Deprivation (SIMD) quintile and the Scottish Government 6 fold urban rural classification (large urban areas, other urban areas, accessible small towns, remote small towns, accessible rural, remote rural).[41 42] The SIMD ranks datazones from the most (number 1) to the least deprived.[41]

Outcome measure

The primary outcome measures were: the client starting a job; and the length of time from beginning the WP to their first job start (months).

Statistical analysis

To address the three research questions a mixture of descriptive statistics and regression analyses were used. All analyses were stratified by benefit type (JSA and ESA clients) given the large differences in RTW between the two groups. Counts and percentages were used to summarise categorical variables. The associations between benefit type and all the study variables were analysed using chi-squared tests. Cox's proportional hazards models were initially used to determine the hazard ratios of

For peer review only - http://bmjopen.bmj.com/site/about/guidelines.xhtml

clients returning to work but the proportional hazards assumption was not met. We therefore approximated the survival model using a piecewise Poisson regression model – equivalent to a Cox model with baseline hazard able to vary between sections.[43] Split times used in the models were as follows: 0 to 3 months, 3 to 6 months, 6 to 9 months, 9 to 12 months and 12 to 24 months (due to sample size a three month average probability is shown for the 12 to 24 month time period). We modelled age as a continuous variable using fractional polynomials; this flexible functional form enabled us to predict the probability of having a job start.[44]

Univariate and multivariable Poisson regression analyses were used to calculate incident rate ratios (IRR) and 95% confidence intervals (95% CI) to examine the associations between job start (RTW) and individual, personal and external factors. The unadjusted models contained age and gender and the adjusted models contained all the variables in the study. Overall differences between the categories are reported and for ordered variables (length of unemployment, highest qualification, number of health conditions disclosed to advisor, SIMD quintiles) a linear trend across categories was also determined. Predicted probabilities of RTW were estimated using post-estimation commands following regression modelling, with illustrative results shown for people aged 25 and 50 years when appropriate. All analyses were carried out using Stata version 14.

Patient/client involvement

No patients/clients were involved in developing the hypothesis, the specific aims or the research questions, nor were they involved in developing plans for design or implementation of the study. No patients were involved in the interpretation of study

For peer review only - http://bmjopen.bmj.com/site/about/guidelines.xhtml

results or write up of the manuscript. Clients did provide feedback on emerging findings at yearly stakeholder meetings and a final study symposium.

For peer review only

For peer review only - http://bmjopen.bmj.com/site/about/guidelines.xhtml

RESULTS

Cohort demographics and job start

Of the SOPIE cohort of 13318 clients, 8996 (68%) were claiming JSA and 4322 (32%) were claiming ESA. Table 1 shows the descriptive statistics of the cohort by benefit type for all variables included in the study. As expected, due to welfare benefit rules, more ESA clients were aged 50 and over (ESA clients 31%; JSA clients 16%). There were statistically significant differences ($p<0.001$) between the two client groups across all variables, except for the test of association between SIMD quintiles ($p<0.05$). The table also shows jobs starts for each variable, with 5612 JSA clients (62%) and 867 ESA clients (20%) having at least one job start during the two-year programme.

The influence of age and job start

Figure 1 shows how the predicted probability of JSA and ESA clients having a job start at any point during the two-year WP varies according to baseline age and gender. There is a strong negative relationship between age and having a job start for both JSA and ESA clients. Male JSA clients have a higher probability of job start compared to female JSA clients at all ages. However, there is no difference in the probability of a job start for male and female ESA clients.

The influence of age and job start during the two-year WP intervention

Figures 2 and 3 show the predicted probability of job start for all four three-month periods in year 1 of the WP, and the average for all three month periods in the whole of year two of the WP, for 25 and 50 year old JSA (Figure 2) and ESA clients (Figure 3), by gender.

For peer review only - http://bmjopen.bmj.com/site/about/guidelines.xhtml

There are marked differences both by age and benefit type when clients are more likely to have a job start throughout the two-year programme. Younger JSA clients have the highest predicted probability of job start in the first three months of their participation in the programme (females 23%, males 28%) which falls off every three months through the programme to 9.6% for females and 11.7% for males in an average three month period in year two of the programme. Older JSA clients have a similar probability of a job start by gender in the first six months of the programme (female 15%, male 18%), which decreases to 5.8% and 6.8% for females and males respectively in an average three month period in year two (Figure 2).

ESA clients, with more health conditions, have a much lower probability of job start compared to JSA clients, particularly in the first three months of the programme (ESA 25 year olds 4%, 50 year olds 2.4%). Apart from a small increase (less than 1%) between four to six months the probability of a job start remains largely unchanged for 25 and 50 year olds ESA clients for the remainder of the programme, with the younger clients having a higher probability (Figure 3).

Factors associated with job start

Unadjusted and multivariable adjusted incident rate ratios (IRR) for factors associated with having a job start for JSA clients are shown in Table 2. Unadjusted analyses showed that female JSA clients were 16% less likely than male JSA clients to have a job start but this association was entirely attenuated when adjusting for all other factors. The length of unemployment before joining the WP was a strong predictor of job start. Clients having educational qualifications under five General Certificate of

For peer review only - http://bmjopen.bmj.com/site/about/guidelines.xhtml

Secondary Education (GCSEs, or equivalent) or below GSCE level were 17% and 23% respectively less likely to have a job start compared to those with a degree or higher. Unadjusted analyses showed that compared to white British clients, other clients were 12% more likely to have a job start (but when adjusted this association was not significant).

In terms of clients' health, disclosing one health condition was associated with a 12% decrease, two health conditions a 24% decrease and three or more health conditions a 45% decrease in the likelihood of having a job start compared to disclosing no health conditions. Having health concerns which the client believed would affect their ability to work versus having no health concerns, was associated with a 36% decrease in the likelihood of having a job start. Client perception of job start was a strong predictor of job start.

In terms of personal circumstances having caring responsibilities for anyone other than children and living in insecure housing (which included temporary housing and homelessness) were associated with a decreased likelihood of job start. Although unadjusted analyses showed significant associations with parental status, when adjusted all associations were lost. In terms of external factors JSA clients living in SIMD quintile 1 were less likely to have a job start, and those living in large urban areas were more likely to have a job start however the associations were lost in the adjusted model.

Unadjusted and multivariable adjusted IRR for factors associated with having a job start for ESA clients are shown in Table 3. Females were as likely as male clients to

For peer review only - http://bmjopen.bmj.com/site/about/guidelines.xhtml

have a job start. Increasing length of unemployment was significantly associated with a lower probability of having a job start. Whilst there was a relationship between education and RTW in the unadjusted model, this disappeared in the fully adjusted model. Non-white British clients were less likely to have a job start.

For ESA clients, disclosing health conditions was associated with a 16% decrease (two health conditions), 32% decrease (three health conditions), 51% decrease (four health conditions) and 49% decrease (five or more health conditions) in the likelihood of having a job start compared to disclosing zero or one health conditions. Having health concerns which the client believed would affect their ability to work versus having no health concerns, was associated with a 34% decrease in the likelihood of having a job start. Clients' perceptions of job start were a strong predictor of job start. Compared to those clients who thought they would start work within 1 month, clients who thought they would start work in more than 4 months were significantly associated with a reduction in job start in the adjusted model. In terms of personal circumstances only parental status was significantly associated with job start, and those with no children less likely to have a job start. In the unadjusted model those clients living in quintiles 2 to 5 were more likely to have a job start than clients living in quintile 1 (most deprived) but this association was lost after adjustment in the full model. ESA clients living in small towns and accessible rural areas were more likely to have a job start than those living in large urban areas, however the effect was lost in the full model.

For peer review only - http://bmjopen.bmj.com/site/about/guidelines.xhtml

DISCUSSION

This study sought to investigate the role of age, health and other factors associated with RTW among two client groups engaging with the two-year WP. There is a strong continuous negative relationship between age and having a job start for both JSA and ESA clients, with no clear evidence for a specific age at which RTW becomes much less likely. For all JSA clients, the probability of a job start is highest in the first three months from joining the programme, decreasing progressively throughout rest of the programme. For ESA clients the probability of a job start changes little throughout the WP. We further identified a range of factors which were associated with JSA and ESA clients having a job start, including some that have not been explored in previous RTW programmes. Our findings discussed below reveal some benefit type similarities as well as some interesting differences with the literature.

Reducing the disability employment gap and enabling more older people to work for longer are key policy challenges.[12 35] This study is therefore particularly important as it extends our understanding of the factors, including age and health as well as certain socio-economic factors, which are associated with RTW and by investigating the JSA and ESA clients separately we have detected further differences which other studies were not able to discern.[8 37] The unique and rich SOPIE cohort also benefits from a two-year follow up on all clients. While several studies have reviewed RTW in welfare-to-work initiatives, these have been limited in several ways, for example they examine fewer explanatory variables and discrete age categories.[8 45 46] Thus, the size of the SOPIE cohort and the range of the variables collected are considerable strengths. While the availability of such rich data on RTW is a major strength of this study, there are also some limitations to the data. The research team

For peer review only - http://bmjopen.bmj.com/site/about/guidelines.xhtml

only had access to the variables routinely collected and could not specify the data collection. Apart from the employment outcomes the baseline data were generally collected when a client was first referred to the programme and data on external factors such as job opportunities is limited. While this study is limited to clients in Scotland the results are generalizable to the rest of the UK.

In terms of the first research question, older age has been shown to be associated with a lower likelihood of RTW, consistent with many other studies.[8 45-49] However, Figures 2 and 3 suggest that while in the first 9 months younger JSA clients have a higher likelihood of RTW, after that period the rates differ little by age. For ESA clients the age differentials remain for the full two years of the programme. For research question 2, the highest predicted probability of job start is in the first three months of the programme for JSA clients. These clients could simply be the most employable clients (e.g. in terms of skills, experience etc.) and so be an example of 'creaming' (where the support provider prioritises those unemployed claimants with fewer barriers to work and who are therefore felt to be easier and more likely to move into paid work) as suggested in other studies.[50 51] In contrast although much lower than JSA clients, the highest predicted probability of job start for the ESA clients (both young and old) was between 4 and 6 months. This is unsurprising given the ESA clients had been unemployed for much longer than JSA clients, had more health conditions (Table 1) and may require more support or time for the modification of health barriers or their perceived redundancy of skills. Both client groups have a decrease in RTW probability after 6 months indicating that increased support or a change in the type of support (including perhaps new approaches) may be required as time in the programme increases, or they may be seen as being so far from the labour

For peer review only - http://bmjopen.bmj.com/site/about/guidelines.xhtml

market employers may not be willing to make sufficient workplace adjustments or feel financial incentives, such as wage subsidies, are needed [52] or consider them as being virtually unemployable in current labour market conditions.[53]

Research question 3 asked what other factors are associated with RTW for JSA and ESA clients. In addition to age, the factors we investigated were drawn from a broad framework of employability covering three main interrelated components, or sets of factors, that influence a person's employability: individual factors, personal circumstances and external factors.[54 55] Although previous studies have shown gender differences in the likelihood of RTW,[8 45-47 56] the gender difference that was observed for JSA clients is lost when all other factors were included in the adjusted model. Those with poorer employment records (especially longer periods of unemployment) on entering the WP were less likely to have a job start and this confirms much prior evidence of having recent work experience prior to claim/entry to programme for RTW.[8 45 46] Our results further confirm earlier evidence that higher qualifications are important in influencing RTW,[46 49] but interestingly not for ESA clients. Whilst there was a relationship between educational qualifications and RTW in the unadjusted model, this disappeared in the fully adjusted model. Further investigation showed this to be due to the relationship between RTW and both length of unemployment and the client's perception of when they saw themselves starting a new job. Ethnicity was important for ESA clients (at the 5% significance level, although not significant for JSA clients) suggesting clients other than white British may require more appropriate support.

For peer review only - http://bmjopen.bmj.com/site/about/guidelines.xhtml

Health is a major obstacle to the re-employment of benefit claimants.[7 8 45 46] With increasing age the prevalence of long term conditions and disability also increases.[57-59] Interestingly we found that 29% of the JSA clients disclosed at least one health condition (50% of the over 50 JSA clients) which would suggest that health is still a potential barrier to RTW for JSA clients and should not be ignored. As with other studies, disclosing a health condition (and the novel finding of increasing number) was significantly associated with the decreased likelihood of a job start.[8 47 48 59] Further, health beliefs were important for both client groups confirming the impact of psychosocial factors. Client perception of their likely job start was important for both client groups and negative expectations may reduce resilience and lead to self-fulfilling outcomes of lower RTW. This is consistent with other studies that have shown that clients' own assessment of their ability to RTW was a strong predictor.[60-62]

Personal circumstances included a range related to individuals' social and household circumstances. These may affect the ability, willingness or social pressure for someone to take up an employment opportunity.[63] For JSA clients, childcare had relatively few effects. Hence the, usually gendered, effects of having dependent children seem limited. However for ESA clients having parental responsibilities actually increased the likelihood of a job start, perhaps due partly to psychosocial factors or a reflection of their level of disability. For non-child caring responsibilities there was a significant impact for JSA clients but the numbers were small (only 4.8% of JSA clients). Housing status was important for JSA clients, although in the adjusted analyses it would appear that it was the clients living in insecure housing that was driving much of this overall negative association. For those in temporary or sheltered

For peer review only - http://bmjopen.bmj.com/site/about/guidelines.xhtml

housing it is difficult to find a job as some employers may prefer those with a more fixed abode or it may be difficult for clients to actually apply of a job if they have no permanent address.[64]

In terms of external factors, as with a WP evaluation,[8] the unadjusted results show that areas of greatest deprivation were associated with lower RTW, but this association was lost in the full models for both client groups. In the unadjusted models those JSA clients living in areas other than large urban areas were less likely to have a job start whereas ESA clients living in small towns and accessible rural areas were more likely to have a job start however these effects disappeared after adjusting for other factors.

It is widely recognised that being employed can improve a person's health and wellbeing and help reduce health inequalities.[2 5] The key findings of this study have important implications for policy makers. While the disability employment gap has been recognised in Government policy,[36] there is little evidence that current programmes will reduce barriers to the employment of ageing workers. While they will provide specialised support for those unemployed for over two years our findings would suggest much earlier intervention is needed. This is also supported by evidence that the longer an individual is absent from work, the less likely it is that they will return, and early intervention for those off work sick has been shown to be effective.[65 66] Programmes for those with health conditions or disabilities are likely to be voluntary, but therefore may not engage individuals who, because of their unemployment, are more likely to have low mood, have an inappropriately pessimistic outlook, be socially isolated and reluctant to access support which needs

For peer review only - http://bmjopen.bmj.com/site/about/guidelines.xhtml

to be based on the biopsychosocial model.[67 68] Perceptions about ability to work are important, but these may have been influenced by health professionals or other advisors with little knowledge of occupational health, workplaces or access to vocational rehabilitation expertise.[65] This study clearly shows that for the individual there is an inverse relationship between job start and the number of health conditions highlighting the need for health care providers to include vocational rehabilitation as part of treatment pathways.[69] Linked to improving workability are education and retraining of ageing workers with medical conditions who may be unfit for their usual role and be disadvantaged because of poor IT or other skills.[15] At present in the UK much of the educational focus has been on the young unemployed,[70] and new programmes need to include training to update and develop new skills for older workers.

While it is generally accepted that most work is beneficial to health,[5] the potential health impacts of engaging with the WP requires further evaluation and linkage of this cohort to National Health Service (NHS) Scotland Information Services Division (ISD) health data is planned. More nuanced estimates of contextual factors such as personal circumstances (including the influence of others in the household) and external factors (such as types of local labour demand, employer behaviours and transport provision), would be useful in refining their influence and importance. Future research also needs to evaluate the longer term vocational outcomes of RTW programmes and whether expected health benefits of RTW are realised by these programmes, particularly when distinguishing the types of jobs people enter and the possible increase in job precariousness and insecurity.

For peer review only - http://bmjopen.bmj.com/site/about/guidelines.xhtml

CONCLUSION

Age, health and a variety of socio-economic factors, play an important role in influencing RTW for unemployed people and for people who have an illness, health condition or disability that makes it difficult to RTW. Other countries with similar types of programmes, supporting both disabled and other job seekers, may also find similar relationships between individual characteristics and personal circumstances of participants. The results from this study will help inform interventions focussing on addressing age-specific, health and biopsychosocial barriers for future RTW programmes with the aim of improving employment outcomes, so that individuals, society, employers and the wider economy can benefit from extending working lives.

For peer review only

For peer review only - http://bmjopen.bmj.com/site/about/guidelines.xhtml

Author Statement

EBM, JB, SVK, AHL, RWM and JF had the original idea and developed the overall research design. JB conducted the statistical analysis with significant advice from SVK, AHL, RWM and JF. JB wrote the first draft. All authors subsequently contributed and commented to the manuscript and approved the final version.

Funding

This project (and researcher JB) is funded by the MRC as part of MRC LLHW Extending Working Lives Partnership Awards (MR/L006367/1). AHL and SVK are funded by the MRC (MC_UU_12017/13 and MC_UU_12017/15) and the Scottish Government Chief Scientist Office (SPHSU13 and SPHSU15). SVK is supported by a Fellowship from the Scottish Government Chief Scientist Office (SCAF/15/02). JF is funded by the MRC (MR/K023209/1) and Scottish Government Chief Scientist Office (SCPHRP), as well as the Usher Institute of Population Health Sciences and Informatics at the University of Edinburgh.

Acknowledgments

The authors would like to acknowledge the help of Paul de Pellette, Luke Jeavons, Cem Zobu at Ingeus, Oarabile Molaodi, Daniel Mackay at the University of Glasgow and the DWP.

Ethics

Ethical approval for this project was received from the University of Glasgow College of Social Sciences Research Ethics Committee (400140186).

For peer review only

For peer review only - http://bmjopen.bmj.com/site/about/guidelines.xhtml

Patient consent

Not required.

Competing interests

None declared.

Data sharing statement

Ingeus acts as data processor on behalf of the data controller, the DWP. Ingeus is able to share data with the University of Glasgow (UoG) as detailed in the MRC Collaboration Agreement and the Research Data Licence between UoG and DWP.

For peer review only

For peer review only - http://bmjopen.bmj.com/site/about/guidelines.xhtml

Figure 1 Predicted probability of JSA and ESA clients having a job start by age during the two-year WP

Figure 2 Step graph showing predicted probability of 25 & 50 year old female and male *JSA* clients having a job start in a 3 month period

Figure 3 Step graph showing predicted probability of 25 & 50 year old female and male *ESA* clients having a job start in a 3 months period

For peer review only

For peer review only - http://bmjopen.bmj.com/site/about/guidelines.xhtml

Table 1 Descriptive characteristics and job start of the SOPIE cohort by benefit type

	JSA clients		ESA clients	
	**No. of clients (% of total JSA clients)**	**No. of clients with job start (% of each category with job start)**	**No. of clients (% of total ESA clients)**	**No. of clients with job start (% of each category with job start)**
**Benefit type**	8996	5612 (62.4%)	4322	867 (20.1%)
**Individual factors**				
**Age**				
Under 50	7590 (84.4%)	4919 (64.8%)	3000 (69.4%)	685 (22.8%)
Over 50	1406 (15.6%)	693 (49.3%)	1322 (30.6%)	182 (13.8%)
**Gender**				
Male	5799 (64.5%)	3754 (64.7%)	2260 (52.3%)	450 (19.9%)
Female	3197 (35.5%)	1858 (58.1%)	2062 (47.7%)	417 (20.2%)
**Length of prior unemployment**				
0-6 months	638 (7.1%)	476 (74.6%)	128 (3.0%)	73 (57.0%)
7-12 months	2034 (22.6%)	1498 (73.7%)	264 (6.1%)	118 (44.7%)
1-2 years	3510 (39.0%)	2416 (68.3%)	733 (17.0%)	261 (35.6%)
3-5 years	1072 (11.9%)	571 (53.3%)	802 (18.6%)	173 (21.6%)
6-10 years	886 (9.9%)	399 (45.0%)	885 (20.5%)	119 (13.5%)
11 years+	856 (9.5%)	252 (29.4%)	1510 (34.9%)	123 (8.2%)
**Highest qualification**				
Degree or higher	580 (6.5%)	436 (75.2%)	165 (3.8%)	64 (38.8%)
A levels/NVQ Level 3 and equivalent	1443 (16.0%)	1006 (69.7%)	480 (11.1%)	149 (31.0%)
5 or more GCSEs Grades A*-C and equivalent	1564 (17.4%)	1094 (70.0%)	468 (10.8%)	134 (28.6%)
Under 5 GCSEs A*-C and equivalent	2145 (23.8%)	1317 (61.4%)	975 (22.6%)	185 (19.0%)
Below GSCE level	3264 (36.3%)	1759 (53.9%)	2234 (51.7%)	335 (15.0%)
**Ethnicity**				

For peer review only

For peer review only - http://bmjopen.bmj.com/site/about/guidelines.xhtml

White British<br>Other	7950 (88.4%)<br>1046 (11.4%)	4906 (61.7%)<br>706 (67.5%)	4062 (94.0%)<br>260 (6.0%)	813 (20.0%)<br>54 (20.8%)
**Have health concerns which believe will affect ability to work**				
No	7247 (80.6%)	4984 (68.8%)	255 (5.9%)	126 (49.4%)
Yes	1749 (19.4%)	628 (35.9%)	4067 (94.1%)	741 (18.2%)
**Number of health conditions disclosed**				
0	6365 (70.8%)	4399 (69.1%)		
0 and 1			1290 (29.8%)	381 (29.5%)
1	1727(19.2%)	905 (52.4%)		
2	608 (6.8%)	239 (39.3%)	1396 (32.3%)	296 (21.2%)
3			896 (20.7%)	123 (13.7%)
3 or more	296 (3.3%)	69 (23.3%)		
4			425 (9.8%)	40 (9.4%)
5 or more			315 (7.3%)	27 (8.6%)
**Client perception of job start - When do you see yourself starting work?**				
Within 1 month	2188 (24.3%)	1646 (75.2%)	92 (2.1%)	56 (60.9%)
2-3 months	3268 (36.3%)	2258 (69.1%)	248 (5.7%)	142 (57.3%)
4-6 months	875 (9.7%)	486 (55.5%)	262 (6.1%)	117 (44.7%)
>6 months	397 (4.4%)	115 (29.0%)	1302 (30.1%)	140 (10.8%)
Don’t know	2268 (25.2%)	1107 (48.8%)	2418 (56.0%)	412 (17.0%)
**Personal circumstances**				
**Caring responsibility for anyone other than children**				
No	8561 (95.2%)	5398 (63.0%)	4046 (93.6%)	822 (20.3%)
Yes	435 (4.8%)	214 (49.2%)	273 (6.4%)	45 (16.3%)
**Housing**				
Homeowner	401 (4.5%)	261 (65.1%)	323 (7.5%)	95 (29.4%)
Living with family	3049 (33.9%)	2155 (69.4%)	609 (14.1%)	146 (24.0%)

For peer review only - http://bmjopen.bmj.com/site/about/guidelines.xhtml

Rented private	1304 (14.5%)	850 (65.2%)	563 (13.0%)	126 (22.4%)
Rented social	3853 (42.8%)	2212 (57.4%)	2655 (61.4%)	469 (17.7%)
Insecure	389 (4.3%)	174 (44.7%)	172 (4.0%)	31 (18.0%)
**Parental status**				
No children	4892 (54.4%)	3218 (65.8%)	1693 (39.2%)	329 (19.4%)
Children, two parent family	622 (6.9%)	406 (65.3%)	288 (6.7%)	89 (30.9%)
Children, shared custody/ not living with you	1254 (13.9%)	735 (58.6%)	711 (16.5%)	145 (20.4%)
Children, lone parent family	1369 (15.2%)	800 (58.4%)	597 (13.8%)	135 (22.6%)
Children, adults living at home/ adults not living at home	859 (9.6%)	453 (52.7)	1033 (23.9%)	169 (16.4%)
**External factors**				
**SIMD quintiles**				
1 (most deprived)	4779 (53.1%)	2899 (60.7%)	2362 (54.7%)	411 (17.4%)
2	2075 (23.1%)	1302 (62.8%)	1013 (23.4%)	219 (21.6%)
3	1151 (12.8%)	707 (61.4%)	550 (12.7%)	130 (23.6%)
4	603 (6.7%)	419 (69.5%)	226 (5.2%)	57 (25.2%)
5 (least deprived)	388 (4.3%)	285 (73.5%)	171 (4.0%)	50 (29.2%)
**6-fold urban rural classification**				
1 Large Urban Areas	4573 (50.8%)	2891 (63.2%)	2578 (59.7%)	467 (18.1%)
2 Other Urban Areas	3176 (35.3%)	1962 (61.8%)	1237 (28.6%)	275 (22.2%)
3 Accessible Small Towns	623 (6.9%)	395 (63.4%)	232 (5.4%)	57 (24.6%)
4 Remote Small Towns	173 (1.9%)	87 (50.3%)	69 (1.6%)	20 (29.0%)
5 Accessible Rural	333 (3.7%)	211 (63.4%)	151 (3.5%)	38 (25.2%)
6 Remote Rural	118 (1.3%)	66 (55.9%)	55 (1.3%)	10 (18.2%)

Test of association on all variables and benefit type, p-value< 0.001, except for test of association on SIMD quintiles and benefit type, p-value<0.05

For peer review only - http://bmjopen.bmj.com/site/about/guidelines.xhtml

For peer review only

For peer review only - http://bmjopen.bmj.com/site/about/guidelines.xhtml

Table 2 Factors associated with job start for *JSA clients*

**Variable**	**Unadjusted IRR (95% CI)***	**p-value**	**Adjusted IRR (95% CI)#**	**p-value**
**Individual factors**				
**Gender**		p<0.001		p=0.435
Male	1.00		1.00	
Female	0.84 (0.80, 0.89)		0.95 (0.89, 1.02)	
**Length of prior unemployment**		p<0.001		p<0.001
0-6 months	1.00		1.00	
7-12 months	0.95 (0.85, 1.05)		0.98 (0.88, 1.09)	
1-2 years	0.80 (0.72, 0.88)		0.84 (0.76, 0.93)	
3-5 years	0.51 (0.45, 0.58)		0.63 (0.56, 0.72)	
6-10 years	0.39 (0.34, 0.44)		0.51 (0.44, 0.58)	
11 years+	0.26 (0.21, 0.28)		0.39 (0.33, 0.45)	
**Highest qualification**		p<0.001		p<0.01
Degree or higher	1.00		1.00	
A levels/NVQ Level 3 and equivalent	0.83 (0.74, 0.92)		0.95 (0.84, 1.06)	
5 or more GCSEs Grades A*-C and equivalent	0.79 (0.71, 0.89)		0.94 (0.84, 1.06)	
Under 5 GCSEs A*-C and equivalent	0.63 (0.56, 0.70)		0.83 (0.74, 0.93)	
Below GSCE level	0.55 (0.49, 0.61)		0.76 (0.68, 0.85)	
**Ethnicity**		p<0.01		p=0.476
White British	1.00		1.00	
Other	1.12 (1.03, 1.21) p<0.01		0.96 (0.88, 1.04)	
**Have health concerns which believe will affect ability to work**		p<0.001		p<0.001
No	1.00		1.00	
Yes	0.41 (0.37, 0.44)		0.64 (0.58, 0.70)	
**Number of health conditions disclosed**		p<0.001		p<0.001
0	1.00		1.00	
1	0.66 (0.61, 0.71)		0.88 (0.82, 0.95)	

For peer review only - http://bmjopen.bmj.com/site/about/guidelines.xhtml

2<br>3 or more	0.46 (0.40, 0.52)<br>0.25 (0.20, 0.32)		0.76 (0.66, 0.88)<br>0.55 (0.43, 0.71)	
**Client perception of job start - When do you see yourself starting work?**<br>Within 1 month<br>2-3 months<br>4-6 months<br>>6 months<br>Don't know	 <br>1.00<br>0.81 (0.76, 0.87)<br>0.55 (0.50, 0.61)<br>0.25 (0.21, 0.31)<br>0.49 (0.45, 0.53)	p<0.001	 <br>1.00<br>0.83 (0.78, 0.89)<br>0.65 (0.58, 0.72)<br>0.41 (0.34, 0.50)<br>0.64 (0.59, 0.69)	p<0.001
**Personal circumstances**				
**Caring responsibility for anyone other than children**<br>No<br>Yes	 <br>1.00<br>0.74 (0.64, 0.85)	p<0.001	 <br>1.00<br>0.75 (0.66, 0.86)	p<0.001
**Housing status**<br>Homeowner<br>Living with family<br>Rented private<br>Rented social<br>Insecure	 1.00<br>0.80 (0.69, 0.92)<br>0.80 (0.69, 0.92)<br>0.67 (0.59, 0.76)<br>0.41 (0.34, 0.50)	p<0.001	 1.00<br>0.99 (0.86, 1.14)<br>1.00 (0.87, 1.16)<br>0.93 (0.81, 1.07)<br>0.65 (0.53, 0.79)	p<0.001
**Parental status**<br>No children<br>Children, two parent family<br>Children, shared custody/ not living with you<br>Children, lone parent family<br>Children, adults living at home/ adults not living at home	 1.00<br>0.98 (0.88, 1.09)<br>0.83 (0.77, 0.91)<br>0.87 (0.79, 0.96)<br>0.99 (0.88, 1.11)	p<0.01	 1.00<br>0.98 (0.87, 1.09)<br>0.93 (0.85, 1.01)<br>1.03 (0.94, 1.14)<br>1.08 (0.96, 1.21)	p=0.505
**External factors**				
**SIMD quintiles**		p<0.001		p=0.145

For peer review only - http://bmjopen.bmj.com/site/about/guidelines.xhtml

1 (most deprived)	1.00		1.00	
2	1.08 (1.01, 1.15)		1.03 (0.97, 1.11)	
3	1.06 (0.98, 1.15)		1.00 (0.92, 1.09)	
4	1.26 (1.14, 1.40)		1.10 (0.99, 1.22)	
5 (least deprived)	1.42 (1.26, 1.61)		1.12 (0.99, 1.28)	
**6-fold urban rural classification**		p<0.05		p=0.069
1 Large Urban Areas	1.00		1.00	
2 Other Urban Areas	0.96 (0.90, 1.01)		0.94 (0.89, 1.00)	
3 Accessible Small Towns	1.00 (0.90, 1.11)		1.02 (0.91, 1.13)	
4 Remote Small Towns	0.66 (0.54, 0.82)		0.73 (0.59, 0.91)	
5 Accessible Rural	0.99 (0.86, 1.14)		0.98 (0.85, 1.13)	
6 Remote Rural	0.82 (0.64, 1.05)		0.76 (0.59, 0.97)	

*Model contained age and gender
Model adjusted for age, gender, length of unemployment, highest qualification, ethnicity, health concerns, number of health conditions, client perception of job start, caring responsibility, housing status, parental status, SIMD quintiles and 6-fold urban rural classification.
Linear trends are shown for length of unemployment, highest qualification, number of health conditions and SIMD quintiles. All other variables show overall trend.

For peer review only

Table 3 Factors associated with job start for *ESA clients*

Variable	Unadjusted IRR (95% CI)*	p-value	Adjusted IRR (95% CI)#	p-value
**Individual factors**				
**Gender**		p=0.632		p=0.141
Male	1.00		1.00	
Female	1.03 (0.90, 1.18)		1.11 (0.95, 1.29)	
**Length of prior unemployment**		p<0.001		p<0.001
0-6 months	1.00		1.00	
7-12 months	0.67 (0.50, 0.90)		0.71 (0.53, 0.96)	
1-2 years	0.49 (0.38, 0.64)		0.57 (0.44, 0.75)	
3-5 years	0.27 (0.20, 0.35)		0.39 (0.29, 0.51)	
6-10 years	0.16 (0.12, 0.21)		0.25 (0.18, 0.33)	
11 years+	0.10 (0.07, 0.13)		0.18 (0.13, 0.25)	
**Highest qualification**		p<0.001		p=0.312
Degree or higher	1.00		1.00	
A levels/NVQ Level 3 and equivalent	0.78 (0.58, 1.05)		1.16 (0.85, 1.58)	
5 or more GCSEs Grades A*-C and equivalent	0.68 (0.51, 0.92)		1.12 (0.82, 1.54)	
Under 5 GCSEs A*-C and equivalent	0.44 (0.33, 0.59)		0.86 (0.63, 1.17)	
Below GSCE level	0.36 (0.27, 0.47)		0.85 (0.64, 1.14)	
**Ethnicity**		p=0.629		p<0.05
White British	1.00		1.00	
Other	1.07 (0.81, 1.41)		0.71 (0.53, 0.95)	
**Have health concerns which believe will affect ability to work**		p<0.000		p<0.000
No	1.00		1.00	
Yes	0.30 (0.25, 0.37)		0.68 (0.55, 0.84) p<0.001	
**Number of health conditions disclosed**		p<0.000		p<0.000
0+1 (Note 121 clients disclosed 0 HCs)	1.00		1.00	

For peer review only - http://bmjopen.bmj.com/site/about/guidelines.xhtml

2	0.68 (0.58, 0.79)		0.84 (0.72, 0.99)	
3	0.44 (0.36, 0.54)		0.67 (0.54, 0.83)	
4	0.30 (0.22, 0.42)		0.49 (0.35, 0.68)	
5 or more	0.28 (0.19, 0.42)		0.52 (0.35, 0.78)	
**Client perception of job start - When do you see yourself starting work?**		p<0.000		p<0.000
Within 1 month	1.00		1.00	
2-3 months	0.81 (0.60, 1.11)		0.97 (0.71, 1.34)	
4-6 months	0.51 (0.37, 0.71)		0.69 (0.49, 0.97)	
>6 months	0.11 (0.08, 0.15)		0.24 (0.17, 0.34)	
Don't know	0.17 (0.13, 0.22)		0.33 (0.24, 0.45)	
**Personal circumstances**				
**Caring responsibility for anyone other than children**		p=0.313		p=0.575
No	1.00		1.00	
Yes	0.86 (0.63, 1.16)		0.89 (0.65, 1.20)	
**Housing status**		p<0.001		p=0.066
Homeowner	1.00		1.00	
Living with family	0.51 (0.38, 0.67)		0.68 (0.50, 0.90)	
Rented private	0.54 (0.41, 0.71)		0.71 (0.53, 0.93)	
Rented social	0.45 (0.36, 0.56)		0.73 (0.58, 0.93)	
Insecure	0.38 (0.25, 0.58)		0.61 (0.40, 0.93)	
**Parental status**		p=0.351		p<0.01
No children	1.00		1.00	
Children, two parent family	1.61 (1.27, 2.04)		1.41 (1.10, 1.80)	
Children, shared custody/ not living with you	1.06 (0.87, 1.29)		1.18 (0.97, 1.45)	
Children, lone parent family	1.12 (0.90, 1.39)		1.25 (0.99, 1.56)	
Children, adults living at home/ adults not living at home	1.13 (0.92, 1.40)		1.26 (1.02, 1.57)	

For peer review only - http://bmjopen.bmj.com/site/about/guidelines.xhtml

External factors				
**SIMD quintiles**		p<0.001		p=0.331
1 (most deprived)	1.00		1.00	
2	1.28 (1.09, 1.51)		1.09 (0.92, 1.29)	
3	1.42 (1.17, 1.75)		0.97 (0.78, 1.19)	
4	1.58 (1.20, 2.09)		1.21 (0.91, 1.61)	
5 (least deprived)	1.86 (1.39, 2.50)		1.17 (0.86, 1.59)	
**6-fold urban rural classification**		p<0.01		p=0.163
1 Large Urban Areas	1.00		1.00	
2 Other Urban Areas	1.22 (1.05, 1.42)		1.04 (0.89, 1.21)	
3 Accessible Small Towns	1.39 (1.06, 1.83)		1.15 (0.87, 1.53)	
4 Remote Small Towns	1.64 (1.05, 2.56)		1.56 (0.98, 2.46)	
5 Accessible Rural	1.51 (1.08, 2.10)		1.08 (0.77, 1.51)	
6 Remote Rural	1.06 (0.57, 1.98)		0.74 (0.39, 1.41)	

*Model contained age and gender

Model adjusted for age, gender, length of unemployment, highest qualification, ethnicity, health concerns, number of health conditions, client perception of job start, caring responsibility, housing status, parental status, SIMD quintiles and 6-fold urban rural classification.

Linear trends are shown for length of unemployment, highest qualification, number of health conditions and SIMD quintiles. All other variables show overall trend.

For peer review only - http://bmjopen.bmj.com/site/about/guidelines.xhtml

References

1. Akinwale B, Lynch K, Wiggins R, et al. Work, permanent sickness and mortality risk: a prospective cohort study of England and Wales, 1971-2006. J Epidemiol Community Health 2011;**65**(9):786-92 doi: 10.1136/jech.2009.099325[published Online First: Epub Date]|.
2. Black C. Working for a healthier tomorrow: Dame Carol Black's Review of the health of Britain's working age population. London, 2008.
3. Katikireddi SV, Leyland AH, McKee M, et al. Patterns of mortality by occupation in the UK, 1991-2011: a comparative analysis of linked census and mortality records. The Lancet Public Health 2017;**2**(11):e501-e12 doi: 10.1016/S2468-2667(17)30193-7[published Online First: Epub Date]|.
4. Katikireddi SV, Niedzwiedz CL, Popham F. Employment status and income as potential mediators of educational inequalities in population mental health. European journal of public health 2016;**26**(5):814-16 doi: 10.1093/eurpub/ckw126[published Online First: Epub Date]|.
5. Waddell G, Burton K. Is work good for your health and wellbeing? The Stationery Office, London, 2006.
6. Bartley M, Sacker A, Clarke P. Employment status, employment conditions, and limiting illness: prospective evidence from the British household panel survey 1991-2001. J Epidemiol Community Health 2004;**58**:501-06
7. Houston D, Lindsay C. Fit for work? Health, employability and challenges for the UK welfare reform agenda. Policy Studies 2010;**31**(2):133-42
8. Meager N, Newton B, Sainsbury R, et al. Work Programme Evaluation: the participant experience report. London, 2014.
9. Thomas C, Benzeval M, Stansfeld S. Employment transitions and mental health: an analysis from the British household panel survey. J Epidemiol Community Health 2005;**59**(3):243-49
10. Altmann R. A New Vision for Older Workers: Retain, Retrain, Recruit. Department for Work and Pensions, London, 2015.
11. Green A, de Hoyos M, Barnes S, et al. Literature review on Employability, Inclusion and ICT, Report 1: The Concept of Employability, with a Specific Focus on Young People, Older Workers and Migrants. Luxembourg, 2013.
12. Department for Work and Pensions. Fuller Working Lives Evidence Base - 2017, 2017.
13. Harper S, Walport M. Future of an Ageing Population. London, 2016.
14. Cappellari L, Dorsett R, Haile G. Labour market transitions among the over-50s. Department for Work & Pensions, London, 2005.
15. Ilmarinen J. Aging workers. Occupational and Environmental Medicine 2001;**58**(8):546-52
16. McNair S. How different is the older labour market? Attitudes to work and retirement among older people in Britain. Social Policy & Society 2006;**5**:485-94
17. Riach M, Loretto W. Identity work and the 'unemployed' worker: age, disability and the lived experience of the older unemployed. Work, employment and society 2009;**23**(1):102-19
18. Canduela J, Dutton M, Johnson S, et al. Ageing, skills and participation in work-related training in Britain: assessing the position of older workers. Work Employ Soc 2012;**26**(1):42-60.
19. Gayen K, McQuaid R, Raeside R. Social networks, age cohorts and employment. International Journal of Sociology and Social Policy 2010;**30**(5/6):219 - 38.

For peer review only - http://bmjopen.bmj.com/site/about/guidelines.xhtml

20. Taylor P, Walker A. The Ageing Workforce: Employers' Attitudes Towards the Employment of Older People. Work Employment & Society 1994; **8**(4):569-91
21. Weir G. The economically inactive who look after the family or home. Labour Market Trends 2002;**110**:577-87
22. Fuertes V, Egdell V, R M. Extending working lives: Age management in SMEs. Employee Relations 2013;**35**(3):272-93
23. Ritchie H, Casebourne J, J R. Understanding workless people and communities: A literature review. Department for Work and Pensions, London, 2005.
24. Mosely H. Decentralisation and Coordination: The Twin Challenges of Labour Market Policy. Organisation for Economic Cooperation and Development, 2008.
25. OECD. Activating Jobseekers: How Australia Does It. Organisation for Economic Cooperation and Development: http://dx.doi.org/10.1787/9789264185920-en 2012.
26. Caswell D, Kleif HB. Disability Pensions and Active Labor Market Policy. Journal of Social Service Research 2013;**39**(4):572-84 doi: 10.1080/01488376.2013.794759[published Online First: Epub Date]|.
27. Finn D. The design of the Work Programme in international context, Report for the National Audit Office. London, 2011.
28. Lantz S, Marston G. Policy, citizenship and governance: the case of disability and employment policy in Australia. Disability & Society 2012;**27**(6):853-67 doi: 10.1080/09687599.2012.686881[published Online First: Epub Date]|.
29. Ulmetsig R. Incapacity benefits - change and continuity in the Swedish welfare state. In: Lindsay C, Houston D, eds. Disability benefits, welfare reform and employment policy. Basingstoke: Palgrave Macmillan, 2013:pp 178-98.
30. Van Berkel R. From Dutch disease to Dutch Fitness? Two decades of disability crisis in the Netherlands. In: Lindsay C, Houston D, eds. Disability benefits, welfare reform and employment policy. Basingstoke: Palgrave Macmillan, 2013:pp 199-215.
31. Department for Work and Pensions. The Work Programme, 2012.
32. Morse A. The Work Programme. National Audit Office, 2014.
33. The UK Government. Jobseeker's Allowance. Secondary Jobseeker's Allowance 2017. https://www.gov.uk/jobseekers-allowance/eligibility [19/09/2017].
34. The UK Government. Employment and Support Allowance. Secondary Employment and Support Allowance 2017. https://www.gov.uk/employment-support-allowance [19/09/2017].
35. Department for Work and Pensions, Department of Health. Improving Lives: The Work, Health and Disability Green Paper, 2016.
36. Department for Work and Pensions, Department for Health. Improving Lives: The Future of Work, Health and Disability, 2017.
37. Foster S, Colechin J, Bivand P, et al. Employment support for unemployed older people, 2014.
38. Age UK. Helping 50+ jobseekers back to work:lessons for the Work and Health Programme, 2016.
39. Brown J, Neary J, Katikireddi SV, et al. Protocol for a mixed-methods longitudinal study to identify factors influencing return to work in the over 50s participating in the UK Work Programme: Supporting Older People into Employment (SOPIE). BMJ Open 2015;**5**(12) doi: 10.1136/bmjopen-2015-010525[published Online First: Epub Date]|.

For peer review only - http://bmjopen.bmj.com/site/about/guidelines.xhtml

40. The Scottish Government. Scottish Neighbourhood Statistics Guide. Secondary Scottish Neighbourhood Statistics Guide. Available from http://www.gov.scot/Publications/2005/02/20697/52626 [05/10/15].
41. The Scottish Government. Scottish Index of Multiple Deprivation. Secondary Scottish Index of Multiple Deprivation. Available from: http://www.gov.scot/Topics/Statistics/SIMD [11/09/15].
42. The Scottish Government. Scottish Government Urban Rural Classification. Secondary Scottish Government Urban Rural Classification. http://www.gov.scot/Topics/Statistics/About/Methodology/UrbanRuralClassification [19/09/17].
43. Laird N, Olivier D. Covariance Analysis of Censored Survival Data Using Log-Linear Analysis Techniques. Journal of the American Statistical Association 1981;**76**(374):231-40 doi: 10.1080/01621459.1981.10477634[published Online First: Epub Date]|.
44. Royston P, Ambler G, Sauerbrei W. The use of fractional polynomials to model continuous risk variables in epidemiology. International Journal of Epidemiology 1999;**28**(5):964-74 doi: 10.1093/ije/28.5.964[published Online First: Epub Date]|.
45. Kemp PA, Davidson J. Employability trajectories among new claimants of Incapacity Benefit. Policy Studies 2010;**31**(2):203-21 doi: 10.1080/01442870903429637[published Online First: Epub Date]|.
46. Sissons P, Barnes H. Getting back to work? Claim trajectories and destinations of Employment and Support Allowance claimants. Journal of Poverty and Social Justice 2013;**21**(3):233-46
47. Cancelliere C, Donovan J, Stochkendahl MJ, et al. Factors affecting return to work after injury or illness: best evidence synthesis of systematic reviews. Chiropractic & manual therapies 2016;**24**(1):32 doi: 10.1186/s12998-016-0113-z[published Online First: Epub Date]|.
48. Ervasti J, Joensuu M, Pentti J, et al. Prognostic factors for return to work after depression-related work disability: A systematic review and meta-analysis. Journal of psychiatric research 2017;**95**:28-36 doi: 10.1016/j.jpsychires.2017.07.024[published Online First: Epub Date]|.
49. Schuring M, Robroek SJW, Otten FWJ, et al. The effect of ill health and socioeconomic status on labor force exit and re-employment: a prospective study with ten years follow-up in the Netherlands. Scand J Work Environ Health 2013;**39**(2):134-43 doi: 10.5271/sjweh.3321[published Online First: Epub Date]|.
50. Carter E, Whitworth A. Creaming and Parking in Quasi-Marketised Welfare-to-Work Schemes: Designed Out Of or Designed In to the UK Work Programme? J Soc Policy 2015;**44**(2):277-96.
51. Rees J, Whitworth A, Carter E. Support for All in the UK Work Programme? Differential Payments, Same Old Problem. Social policy & administration 2014;**48**(2):221-39 doi: 10.1111/spol.12058[published Online First: Epub Date]|.
52. Clayton S, Barr B, Nylen L, et al. Effectiveness of return-to-work interventions for disabled people: a systematic review of government initiatives focused on changing the behaviour of employers. European journal of public health 2012;**22**(3):434-9 doi: 10.1093/eurpub/ckr101[published Online First: Epub Date]|.

For peer review only - http://bmjopen.bmj.com/site/about/guidelines.xhtml

53. Saint-Paul G. Are the unemployed unemployable? European Economic Review 1996;**40**:1501-19
54. Koen J, Klehe U-C, Van Vianen A. Employability among the long-term unemployed: A futile quest or worth the effort? Journal of Vocational Behavior 2013;**82**:37-48
55. McQuaid RW, Lindsay C. The Concept of Employability. Urban Studies 2005;**42**(2):197-219
56. Beatty C, Fothergill S, Houston D, et al. Bringing Incapacity Benefit numbers down: to what extent do women need a different approach? Policy Studies 2010;**31**(2):143-62
57. Clayton S, Bambra C, Gosling R, et al. Assembling the evidence jigsaw: insights from a systematic review of UK studies of individual-focused return to work initiatives for disabled and long-term ill people. BMC Public Health 2011;**11**:170 doi: 10.1186/1471-2458-11-170[published Online First: Epub Date]|.
58. Department of Health. Long Term Conditions Compendium of Information: Third Edition, 2012.
59. Lindsay C, Greve B, Cabras I, et al. Assessing the evidence base in health, employability and the labour market - lessons for activation in the UK. 49 2015;**2**:143-60
60. McQuaid RW, Bond S, Fuertes V. Working for Families evaluation. Scottish Government, Edinburgh, 2009.
61. Nieuwenhuijsen K, Noordik E, van Dijk FJH, et al. Return to Work Perceptions and Actual Return to Work in Workers with Common Mental Disorders. J Occup Rehabil 2013;**23**(2):290-99 doi: 10.1007/s10926-012-9389-6[published Online First: Epub Date]|.
62. Wilford J, McMahon AD, Peters J, et al. Predicting job loss in those off sick. Occupational medicine (Oxford, England) 2008;**58**(2):99-106 doi: 10.1093/occmed/kqm141[published Online First: Epub Date]|.
63. Nauta A, van Vianen A, van der Heijden B, et al. Understanding the factors that promote employability orientation: The impact of employability culture, career satisfaction, and role breadth self-efficacy. Journal of Occupational and Organizational Psychology 2009;**82**(2):233-51 doi: 10.1348/096317908x320147[published Online First: Epub Date]|.
64. Ahmed A, Granberg M, Lång E. Does having one's own place to live make someone more employable? Applied Economics Letters 2017;**24**(18):1327-30 doi: 10.1080/13504851.2016.1276263[published Online First: Epub Date]|.
65. Black C, Frost D. Health at work - an independent review of sickness absence. Secondary Health at work - an independent review of sickness absence 2011 2011.
66. Brown J, Mackay D, Demou E, et al. The EASY (Early Access to Support for You) sickness absence service: a four-year evaluation of the impact on absenteeism. Scand J Work Environ Health 2015;**41**(2):204-15 doi: 10.5271/sjweh.3480[published Online First: Epub Date]|.
67. Demou E, Gibson I, Macdonald EB. Identification of the factors associated with outcomes in a Condition Management Programme. Bmc Public Health 2012;**12**:10 doi: 10.1186/1471-2458-12-927[published Online First: Epub Date]|.
68. Engel G. The need for a new medical model: a challenge for biomedicine. Science 1977;**196**:129-36

For peer review only - http://bmjopen.bmj.com/site/about/guidelines.xhtml

69. Health Works. A review of the Scottish Government's Healthy Working Lives Strategy. Secondary Health Works. A review of the Scottish Government's Healthy Working Lives Strategy 2013 2013.
70. Maguire S. NEET, unemployed, inactive or unknown – why does it matter? Educational Research 2015;**57**(2):121-32 doi: 10.1080/00131881.2015.1030850[published Online First: Epub Date]|.

For peer review only

For peer review only - http://bmjopen.bmj.com/site/about/guidelines.xhtml

For peer review only

For peer review only - http://bmjopen.bmj.com/site/about/guidelines.xhtml

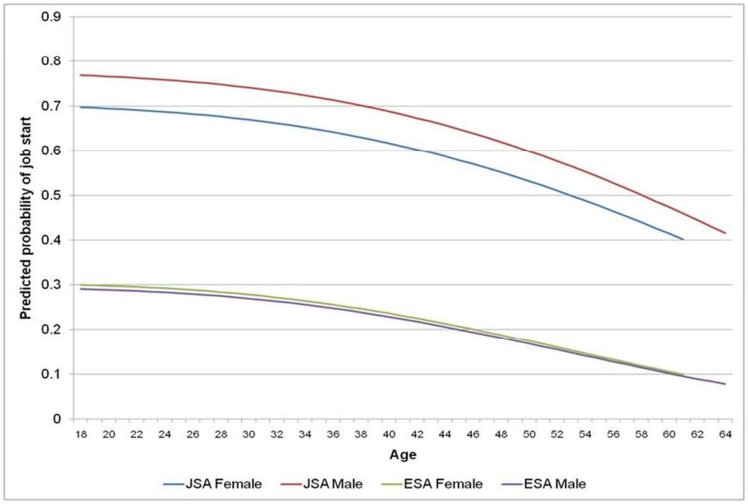

Predicted probability of JSA and ESA clients having a job start by age during the two-year WP

108x60mm (300 x 300 DPI)

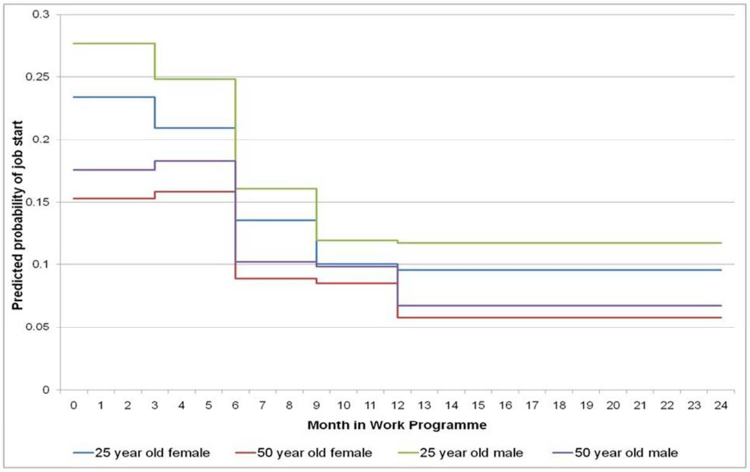

Step graph showing predicted probability of 25 & 50 year old female and male JSA clients having a job start in a 3 month period

108x60mm (300 x 300 DPI)

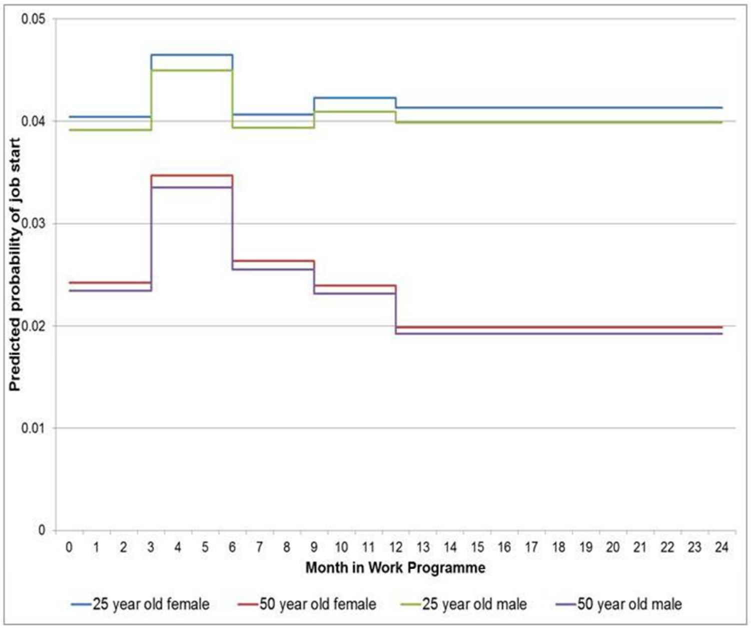

Step graph showing predicted probability of 25 & 50 year old female and male ESA clients having a job start in a 3 months period

81x60mm (300 x 300 DPI)

w only

STROBE 2007 (v4) Statement—Checklist of items that should be included in reports of *cohort studies*

**Section/Topic**	**Item #**	**Recommendation**	**Reported on page #**
**Title and abstract**	1	(*a*) Indicate the study's design with a commonly used term in the title or the abstract	1
		(*b*) Provide in the abstract an informative and balanced summary of what was done and what was found	2 & 3
**Introduction**			
Background/rationale	2	Explain the scientific background and rationale for the investigation being reported	5
Objectives	3	State specific objectives, including any prespecified hypotheses	8
**Methods**			
Study design	4	Present key elements of study design early in the paper	10
Setting	5	Describe the setting, locations, and relevant dates, including periods of recruitment, exposure, follow-up, and data collection	10
Participants	6	(*a*) Give the eligibility criteria, and the sources and methods of selection of participants. Describe methods of follow-up	10
		(*b*) For matched studies, give matching criteria and number of exposed and unexposed	N/A
Variables	7	Clearly define all outcomes, exposures, predictors, potential confounders, and effect modifiers. Give diagnostic criteria, if applicable	10 & 11
Data sources/ measurement	8*	For each variable of interest, give sources of data and details of methods of assessment (measurement). Describe comparability of assessment methods if there is more than one group	10 & 11
Bias	9	Describe any efforts to address potential sources of bias	No specific biases expected. The data was based on 100% sample of Ingeus Work Programme clients. Note that Ingeus clients covered 50% of all Scottish WP clients and were allocated randomly to the Ingeus WP by DWP.
Study size	10	Explain how the study size was arrived at	10 and Protocol

For peer review only

			paper (reference 32)
Quantitative variables	11	Explain how quantitative variables were handled in the analyses. If applicable, describe which groupings were chosen and why	10, 11 & 27 (Table 1)
Statistical methods	12	(*a*) Describe all statistical methods, including those used to control for confounding	11 & 12
		(*b*) Describe any methods used to examine subgroups and interactions	11
		(*c*) Explain how missing data were addressed	10. All missing data (including age) was removed at data cleaning stage.
		(*d*) If applicable, explain how loss to follow-up was addressed	N/A
		(*e*) Describe any sensitivity analyses	N/A
**Results**			
Participants	13*	(a) Report numbers of individuals at each stage of study—eg numbers potentially eligible, examined for eligibility, confirmed eligible, included in the study, completing follow-up, and analysed	After data cleaning 13318 clients, all with follow up
		(b) Give reasons for non-participation at each stage	N/A
		(c) Consider use of a flow diagram	We describe the clients we removed on p10 and is straightforward so don't think a flow diagram is needed.
Descriptive data	14*	(a) Give characteristics of study participants (eg demographic, clinical, social) and information on exposures and potential confounders	13 & 27 (Table 1)
		(b) Indicate number of participants with missing data for each variable of interest	The 947 clients with missing data represented 6.6% of the original cohort. Missing age n=693, postcode (and therefore external factors) n=100, < age 18 n=7, length of unemployment not possible n=147

For peer review only

		(c) Summarise follow-up time (eg, average and total amount)	13
Outcome data	15*	Report numbers of outcome events or summary measures over time	13 & 14
Main results	16	(*a*) Give unadjusted estimates and, if applicable, confounder-adjusted estimates and their precision (eg, 95% confidence interval). Make clear which confounders were adjusted for and why they were included	14, 15, 31 (Table 2) , 34 (Table 3)
		(*b*) Report category boundaries when continuous variables were categorized	We followed outline of good practice.[1]We use deciles for SIMD data and continuous data for age (where the dichotomised ages were presented they were based on theory, empirical papers and the results presented earlier in the paper). The ESA v JSA dichotomy reflected the population who were enrolled in these.
		(*c*) If relevant, consider translating estimates of relative risk into absolute risk for a meaningful time period	N/A
Other analyses	17	Report other analyses done—eg analyses of subgroups and interactions, and sensitivity analyses	N/A
**Discussion**			
Key results	18	Summarise key results with reference to study objectives	17
**Limitations**			
Interpretation	20	Give a cautious overall interpretation of results considering objectives, limitations, multiplicity of analyses, results from similar studies, and other relevant evidence	17
Generalisability	21	Discuss the generalisability (external validity) of the study results	18
**Other information**			
Funding	22	Give the source of funding and the role of the funders for the present study and, if applicable, for the original study on which the present article is based	24

For peer review only

*Give information separately for cases and controls in case-control studies and, if applicable, for exposed and unexposed groups in cohort and cross-sectional studies.

Note: An Explanation and Elaboration article discusses each checklist item and gives methodological background and published examples of transparent reporting. The STROBE checklist is best used in conjunction with this article (freely available on the Web sites of PLoS Medicine at http://www.plosmedicine.org/, Annals of Internal Medicine at http://www.annals.org/, and Epidemiology at http://www.epidem.com/). Information on the STROBE Initiative is available at www.strobe-statement.org.

1. Turner EL, Dobson JE, Pocock SJ. Categorisation of continuous risk factors in epidemiological publications: a survey of current practice. Epidemiologic perspectives & innovations : EP+I 2010;**7**:9 doi: 10.1186/1742-5573-7-9[published Online First: Epub Date].

For peer review only
